# Microbial Lignin Valorization to Protocatechuic Acid and Catechol: Biofunneling Pathways and Metabolic Engineering Strategies

**DOI:** 10.3390/biom16070979

**Published:** 2026-07-03

**Authors:** Yoganathan Kamaraj, Shehbaz Ali, Sethupathy Sivasamy, Mudasir A. Dar, Gao Le, Abida Rani, Veenayohini Kumaresan, Naveed Ahmad, Daochen Zhu

**Affiliations:** 1 Biofuels Institute, School of the Environment and Safety Engineering, Jiangsu University, Zhenjiang 212013, China; yoganathan39@gmail.com (Y.K.); shehbaz205@gmail.com (S.A.); sethulifescience@gmail.com (S.S.); muddar7@ujs.edu.cn (M.A.D.); 2 Jiangsu Collaborative Innovation Center of Technology and Material of Water Treatment, Suzhou University of Science and Technology, Suzhou 215009, China; 3 Post Graduate and Research Department of Biotechnology, Microbiology & Bioinformatics, National College, Tiruchirappalli 620001, Tamil Nadu, India; 4 Department of Biotechnology, Faculty of Sciences, International Institute of Science, Arts and Technology (IISAT), Gujranwala 52250, Pakistan; 5 Clinical Medical College, Hainan Medical University, Haikou 571199, China; 6 Key Laboratory of Engineering Biology for Low-Carbon Manufacturing, Tianjin Institute of Industrial Biotechnology, Chinese Academy of Sciences, 32 West 7th Avenue, Tianjin 300308, China; gao_l@tib.cas.cn; 7 Department of Pharmacy, Faculty of Pharmacy, International Institute of Science, Arts, and Technology (IISAT), Gujranwala 52250, Pakistan; drabida.rani@gmail.com; 8 Department of Biotechnology and Bioinformatics, Holy Cross College, Tiruchirappalli 620002, Tamil Nadu, India; kumsveena@gmail.com; 9 Institute for Safflower Industry Research of Shihezi University, Pharmacy College of Shihezi University, Key Laboratory of Xinjiang Phytomedicine Resource and Utilization, Ministry of Education, Shihezi 832003, China

**Keywords:** lignin, aromatic transporters, protocatechuate decarboxylase, catechol

## Abstract

Lignin, an abundant and renewable aromatic biopolymer, represents a largely underutilized resource for the sustainable production of high-value chemicals. Among lignin-derived intermediates, protocatechuic acid (PCA) and catechol have emerged as key platform molecules due to their versatile applications in pharmaceuticals, polymers, and fine chemicals. This review provides a critical overview of microbial lignin valorization focusing on the microbial conversion of lignin-derived aromatics into PCA and catechol. It highlights recent advances in lignin depolymerization techniques, including thermochemical and biological approaches, and examines their influence on the generation of bioavailable aromatic feedstocks. We systematically discuss microbial biofunneling pathways that converge diverse lignin-derived compounds into PCA and catechol, emphasizing the role of central metabolic nodes and enzymatic transformations such as O-demethylation, hydroxylation, and decarboxylation. We treat protocatechuate decarboxylase (PCADC) as the central enzymatic bridge linking PCA and catechol. However, it should be noted that many reported microbial production strategies have been demonstrated using purified lignin-derived aromatic model compounds (e.g., ferulate, vanillate, *p*-coumarate, and PCA) rather than authentic lignin streams, highlighting the need for improved integration of lignin depolymerization and downstream bioconversion processes. Furthermore, the review explores state-of-the-art metabolic engineering strategies, including gene deletions, pathway rewiring, transporter engineering, and CRISPR-based regulation, to enhance product yields and selectivity. Despite significant progress, several challenges persist, including lignin recalcitrance, heterogeneity of depolymerization products, toxicity of intermediates, and limited enzyme efficiency. This review identifies key knowledge gaps and proposes future directions for integrating synthetic biology, adaptive evolution, and systems-level optimization to develop robust microbial cell factories. Overall, this work provides a strategic framework for advancing lignin bioconversion into PCA and catechol, contributing to the development of sustainable biorefineries and a circular bioeconomy.

## 1. Introduction

Lignocellulosic biomass, a copious and renewable organic carbon reservoir, holds immense potential as a viable substitute for biofuels, biochemicals, and biomolecules. It comprises cellulose, hemicellulose, and lignin. Among these components, lignin plays a significant role in protecting the structural integrity of plant cell walls. Its abundance ranges from 15–30% of plant biomass, and about 140 million tonnes of lignin is released as a byproduct from the pulping, paper, and lignocellulosic biorefinery industries [1]. As the world seeks sustainable and renewable resources, lignin presents an attractive raw material due to its availability and potential for conversion into various high-value products [2,3]. However, the degradation of lignin is still challenging due to its complex and heterogenic nature [4].

Integrated chemical and biological methods have emerged as promising strategies for transforming lignin into valuable products [5,6]. The use of wild and engineered microbial strains for the conversion of these lignin-derived aromatics can result in the production of various beneficial and platform chemicals, such as Protocatechuic acid (PCA) and catechol, which are widely utilized for industrial and biomedical applications [7,8,9,10]. Similar to other techniques, microbial strains also lack potential systems for lignin valorization. However, by strategically addressing these challenges through innovative strain engineering and advanced downstream processing, we can unlock microbial valorization’s full potential for selectively producing PCA and catechol [11].

This review extends beyond earlier assessments that focused mainly on depolymerization and on the microbial conversion of lignin-derived compounds into valuable biochemicals [12,13]. However, the central role of PCA and catechol as convergent intermediates in lignin-derived aromatic metabolism has received comparatively limited attention. Despite the structural diversity of lignin-derived monomers, most well-characterized microbial biofunneling pathways converge toward PCA, catechol, or closely related intermediates such as gallate, highlighting the importance of these compounds as metabolic hubs linking lignin depolymerization with downstream bioproduct synthesis [14].

This review focuses on the PCA–catechol junction, a metabolic framework that integrates lignin feedstock composition, microbial biofunneling pathways, and metabolic engineering strategies for aromatic chemical production. Particular attention is given to the role of protocatechuate decarboxylase (PCADC), which enables the conversion of PCA into catechol and thereby connects two major aromatic metabolic nodes. We propose that efficient lignin valorization depends on the coordinated optimization of feedstock selection, microbial chassis, and target product pathways. By organizing recent advances around this PCA–catechol axis, this review provides a pathway-centric perspective that differs from previous lignin valorization reviews and identifies emerging opportunities for engineering robust microbial cell factories for sustainable aromatic biomanufacturing.

## 2. Protocatechuic Acid and Catechol: Central Nodes in Aromatic Biochemistry and Industrial Biotechnology

**Figure 1:**
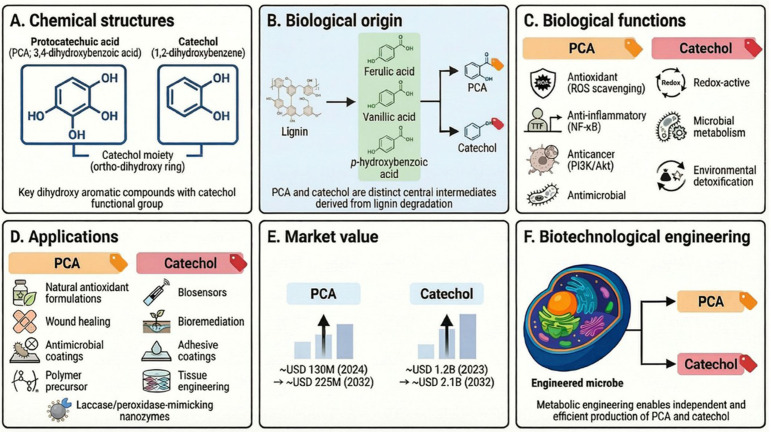
PCA and catechol as central nodes in lignin valorization. (**A**) Chemical structure of PCA and catechol and (**B**) biological origin, (**C**) biological functions, (**D**) industrial applications, (**E**) current and expected global market values [19,20] and (**F**) metabolic engineering opportunities.

Phenolic compounds are a vast class of secondary metabolites abundantly found in plants, and they play crucial roles in both plant physiology and human health. Among them, protocatechuic acid (PCA:3,4-dihydroxybenzoic acid) and catechol (1,2-dihydroxybenzene) are well-studied low-molecular-weight phenolics, distinguished by their catechol moiety, a benzene ring bearing two hydroxyl groups in the ortho position. These compounds are widely distributed in nature, are biosynthetically interconnected, and exhibit diverse biological functions ranging from antioxidant activity to participation in microbial metabolism and environmental bioremediation [15]. PCA and catechol are two crucial aromatic intermediates derived from the microbial degradation of lignin-derived phenolic compounds and other aromatic hydrocarbons. These compounds represent vital biochemical nodes that bridge lignin depolymerization products to a range of industrially and medically valuable metabolites [16,17]. Owing to their simple dihydroxy-substituted aromatic ring structures, PCA and catechol serve as versatile building blocks for the synthesis of numerous high-value biochemicals, including polymers, pharmaceuticals, and fine chemicals [18]. According to market reports, the global PCA market—valued at ≈ USD 130 M in 2024 is expected to reach ≈ USD 225 M by 2032 (CAGR ~ 8.2%). In contrast, catechol already commands a multi-hundred-million to multi-billion-dollar global market (e.g., ≈ USD 1.2 B in 2023, projected to ≈ USD 2.1 B by 2032 [19,20]. In recent years, advances in metabolic engineering and synthetic biology have significantly enhanced microbial pathways for PCA and catechol production, transforming them into key platform molecules for sustainable biotechnological applications (Figure 1) [21,22].

During microbial valorization of lignin, PCA is generated through peripheral funneling of lignin-derived aromatic compounds—such as vanillic acid, p-hydroxybenzoic acid, and ferulic acid—primarily via O-demethylation and hydroxylation reactions. PCA is subsequently catabolized through the β-ketoadipate pathway, whereas its de novo biosynthesis proceeds through the shikimate pathway in many hosts [11,14]. This compound exhibits remarkable pharmacological potential, acting as a potent antioxidant, anti-inflammatory, antimicrobial, and anticancer agent [18,23,24,25,26,27]. It scavenges reactive oxygen species (ROS) and modulates molecular signaling pathways such as NF-κB and PI3K/Akt, leading to cytoprotective and anticancer outcomes [28]. Due to these bioactivities, PCA has attracted increasing interest in developing natural antioxidant formulations, wound-healing agents, antimicrobial coatings, and antitumor therapeutics. From a biotechnological standpoint, PCA is also gaining attention as a precursor for the synthesis of renewable polymers, plasticizers, nanomaterials, and dyes, owing to its aromatic structure and hydroxyl reactivity [29,30]. Beyond their industrial and biomedical applications, PCA and other lignin-derived phenolic compounds have emerged as valuable targets for biosensor development [31,32]. In addition, lignin-derived aromatic compounds and lignin-based nanomaterials are increasingly being explored in electrochemical, fluorescence, and whole-cell biosensing platforms for environmental monitoring, metabolic engineering, and synthetic biology applications [33,34,35].

Catechol, on the other hand, plays a dual role in biotechnology—as both a central metabolic intermediate and a valuable industrial compound [36]. It is a crucial node in the microbial degradation of aromatic pollutants, such as phenol, benzoate, and toluene, through the ortho-cleavage (catechol 1,2-dioxygenase) and meta-cleavage (catechol 2,3-dioxygenase) pathways [37]. These dioxygenase-mediated reactions convert catechol into tricarboxylic acid (TCA) cycle intermediates, completing aromatic carbon assimilation [38]. The enzymatic and metabolic versatility of catechol has also inspired its use in biosensing, bioremediation, and biomaterial design [39]. Catechol-based coatings, inspired by mussel adhesive proteins, are increasingly used in tissue engineering and surface modification of biomedical devices [40,41]. In parallel, catechol’s redox properties make it an excellent substrate for Perovskite Tandem Solar Cells, biosensors targeting phenolic pollutants, neurotransmitters like dopamine, and reactive oxygen species, with laccase-mimicking and peroxidase-mimicking nanozymes now widely utilized for such detection [42,43,44,45].

In microbial and industrial processes, PCA and catechol share a tight biochemical relationship. Both can be derived from lignin-based feedstocks through oxidative depolymerization and subsequent bio-funneling steps, highlighting their significance as sustainable chemical precursors in lignocellulosic biorefineries [1]. The combination of chemical pretreatment, enzymatic depolymerization, and microbial valorization offers an integrated route to generate these aromatic compounds efficiently and selectively. Furthermore, metabolic engineering of model microorganisms such as *Escherichia coli*, *Corynebacterium glutamicum*, and *Pseudomonas putida* has enhanced the flux through aromatic degradation pathways, allowing higher titers of PCA and catechol to be achieved from renewable biomass [22,46]. Metabolic engineering strategies, including the use of CRISPR-based gene editing and protein stability enhancement, further facilitate efficient conversion of heterogeneous lignin streams into valuable PCA and catechol [47].

Recent breakthroughs in metabolic engineering have unlocked new opportunities to convert PCA and catechol into a broad portfolio of industrially valuable chemicals using lignin as a sustainable feedstock. By modifying the native catabolic pathways in bacteria, PCA and catechol metabolism can be redirected toward the synthesis of high-value biochemicals such as β-ketoadipate, *cis,cis*-muconic acid (cc-MA), and 2-pyrone-4,6-dicarboxylic acid (PDC) [48,49,50,51,52], which serve as precursors for bioplastics, synthetic fibers, bio-based resins, and green solvents. In particular, deletion of key ring-cleaving enzymes (e.g., *pcaHG*, *catA*/*A2*) prevents complete aromatic mineralization, enabling intracellular accumulation of PCA and catechol, while overexpression of upper-pathway enzymes such as vanillate O-demethylase (*vanAB*) enhances metabolic funneling of diverse lignin-derived aromatics into these pivotal intermediates [1,53,54].

These pathway engineering strategies—combined with advancements in enzyme optimization, redox balancing, transporter engineering, and CRISPR-enabled regulation—demonstrate the strong biotechnological potential of PCA and catechol as platform molecules [47]. Overall, engineering the PCA and catechol metabolic junction offers a scalable and eco-efficient route to transform lignin-rich biomass into commercially relevant aromatic building blocks, reinforcing the importance of microbial lignin valorization in a circular bioeconomy [55]. Collectively, PCA and catechol stand out as pivotal molecules in the context of lignin valorization. Their production exemplifies the successful convergence of biochemistry, metabolic engineering, and green chemistry principles for sustainable industrial biotechnology. Understanding the enzymatic routes, genetic regulation, and process engineering that govern their biosynthesis will be critical to developing scalable and economically viable bioprocesses.

## 3. From Complexity to Opportunity: Bottlenecks in Lignin-to-Aromatics Conversion

### 3.1. Challenges in Non-Biological Depolymerization Methods

Non-biological lignin depolymerization approaches, particularly thermal and chemical strategies, continue to dominate industrial implementation due to their process robustness, compatibility with large-scale reactors, and rapid reaction kinetics. Thermochemical processes such as pyrolysis, hydrogenolysis, and hydrothermal treatments cleave lignin’s ether and carbon–carbon linkages under high temperatures, sometimes aided by catalysts. Pyrolysis—performed in oxygen-limited conditions produces aromatic monomers and phenolic oils and is considered a promising route for converting lignin into bio-based chemicals [56]. The process typically initiates with the cleavage of weaker β-O-4 bonds, followed by breakdown of stronger linkages at temperatures exceeding 450 °C [57]. Catalytic pyrolysis and solvent-assisted pyrolysis can further enhance product selectivity and reduce char formation.

Microwave-assisted depolymerization has emerged as an energy-efficient alternative to conventional heating, enabling rapid, uniform heating of lignin. Microwave-based methods offer operational advantages such as reduced reaction times, simple reactor design, and improved control over reaction kinetics [58]. In some cases, microwave pyrolysis has yielded high-quality activated carbon and aromatics from biomass [59]. Chemical methods use acids, bases, metals, ionic liquids, or supercritical fluids: acids target ether linkages, alkalis such as NaOH solubilize lignin and break β-O-4 bonds [60], ionic liquids enable selective depolymerization to monomers such as benzoquinones [61], and supercritical fluids enhance mass transfer; oxidative systems using H_2_O_2_ or metal catalysts give oxidized monomers under milder conditions [62,63].

These processes typically generate complex and heterogeneous product mixtures containing inhibitory byproducts, which reduce microbial conversion efficiency [64]. In particular, phenolic-rich oils obtained from pyrolysis exhibit strong cytotoxicity toward engineered microbial strains. Moreover, the monomer profiles produced are often inconsistent, leading to unpredictable biofunneling toward PCA or catechol [14,65]. Their high temperatures and pressures also raise energy and operating costs, limiting scalability. Future work should therefore improve selectivity toward bioavailable monomers (e.g., vanillin, p-hydroxybenzoic acid, ferulate) and develop hybrid chemo-biological strategies that couple depolymerization directly with microbial upgrading.

### 3.2. Challenges in Biological Depolymerization of Lignin

Biological depolymerization is increasingly favoured because it operates under mild conditions, avoids toxic byproducts, and can be coupled directly with microbial funneling pathways. While fungal lignin degradation has been extensively studied since the early 1900s [66,67], bacteria are increasingly recognized for their robustness, adaptability, and potential for industrial-scale valorization. A diverse range of bacteria—including *Serratia*, *Paenibacillus*, *Bacillus*, *Pseudomonas*, *Rhodococcus*, and extremophiles—have shown the capacity to degrade native and technical lignins [68,69]. Many are isolated from aromatic-rich environments such as pulp-mill effluents, compost, rumen, or contaminated soils. Aerobic, anaerobic, and facultative bacteria use distinct enzymatic strategies across oxygen-rich and oxygen-limited conditions [70,71].

Although bacteria contribute to lignin biodegradation in nature, their application for biorefinery processes is constrained by several challenges. Compared with fungi, bacterial lignin depolymerization rates are typically slow, resulting in limited release of monomers required for PCA or catechol biosynthetic pathways. For example, white-rot fungi such as *Phanerochaete chrysosporium* and *Phlebia acerina* can achieve 44–47% lignin weight loss from oat straw within 28 days, and up to 55% from wheat straw in 35 days with *Ceriporiopsis subvermispora*; in direct comparative assays using nitrated milled-wood lignin, *P. chrysosporium* showed 20- to 100-fold higher extracellular lignin-degrading activity than the best-performing bacteria [72]. Typical bacterial isolates, by contrast, achieve 16–34% lignin degradation over 7–15 days—for instance, *Lysinibacillus sphaericus* (24% in 7 days) and *Burkholderia* sp. (16.7% in 15 days) on milled pine—although select strains such as *Brevibacillus thermoruber* can reach 82% under optimised thermophilic conditions [73]. Moreover, many bacterial strains produce broad and heterogeneous aromatic mixtures with low selectivity, complicating downstream metabolic channeling [74]. Depolymerization is often incomplete, leaving substantial high-molecular-weight residues untreated. Additionally, maintaining efficient bacterial activity in high-solids environments or industrial lignin waste streams remains difficult, hindering process scalability [75]. To overcome these limitations, future efforts should emphasize systematic strain discovery, adaptive laboratory evolution, and genomic/metabolic engineering to enhance monomer specificity and ensure better integration with microbial pathways for targeted PCA and catechol production.

Bacteria deploy oxidative and hydrolytic enzymes to cleave lignin’s complex polymeric structure. Key enzyme families include laccases, lignin peroxidases (LiP), manganese peroxidases (MnP), dye-decolorizing peroxidases (DyPs), β-etherases, and cytochrome P450 monooxygenases. Laccases oxidize phenolic lignin structures and can depolymerize both phenolic and non-phenolic units when coupled with redox mediators. Bacterial laccases have been identified in species such as *Bacillus altitudinis* and *Bhargavaea beijingensis*, expanding their relevance for lignin valorization [76,77]. LiP and MnP generate radicals that cleave interunit bonds, as in *B. ligniniphilus* L1 and *B. thermoruber* [78,79]. DyP-type peroxidases, common in bacteria, exhibit strong activity toward β-O-4 linkages and produce monomers like veratraldehyde [80,81]. Cytochrome P450 systems introduce hydroxylation and demethylation reactions, further modifying lignin aromatics for funneling [82]. Hydrolytic enzymes such as β-etherases cleave the dominant β-O-4 ether bond and may act synergistically with oxygenases and peroxidases. Together these convert lignin into aromatic aldehydes, acids, and alcohols that feed central intermediates—PCA, catechol, gallic acid, and vanillic acid—which then enter the TCA cycle as acetyl-CoA, succinyl-CoA, and pyruvate.

Nevertheless, significant challenges remain before bacterial lignin depolymerization can be routinely deployed at scale: degradation rates are typically slow, product yields remain low, and monomer specificity is often poor—resulting in heterogeneous aromatic mixtures that are ill-suited for efficient funneling into target compounds [83]. Looking forward, systematic approaches such as bioprospecting for novel robust strains, adaptive-laboratory evolution, and metabolic/enzyme engineering (e.g., to improve enzyme stability, activity, and monomer selectivity) coupled with modern synthetic-biology tools may unlock the potential of bacteria to generate high-value lignin-derived monomers in a controlled, scalable, and economically viable manner [55].

### 3.3. Influence of Lignin Source on Monomer Composition and Feedstock Suitability

The type of biomass feedstock fundamentally determines the aromatic monomer slate released upon depolymerization and, consequently, the efficiency with which it can be funneled to PCA or catechol. Native lignin is assembled from three monolignols—p-coumaryl, coniferyl, and sinapyl alcohol—that give rise to p-hydroxyphenyl (H), guaiacyl (G), and syringyl (S) units, respectively, and the relative abundance of these units varies markedly with plant source [84]. Softwood (gymnosperm) lignin is composed almost entirely of G units (~80–90%) with trace H and essentially no S units (S/G ≈ 0.01–0.02 in pine and spruce); hardwood (angiosperm) lignin contains both G (~25–50%) and S (~50–70%) units (S/G up to ~2 in birch); and herbaceous/grass lignin incorporates all three (G, S, and H) units and is additionally enriched in ester-linked hydroxycinnamates, namely *p*-coumarate and ferulate [84].

These compositional differences translate directly into different bioconversion outcomes. G-derived monomers (vanillin, vanillate, guaiacol, coniferyl-type compounds) are readily channeled to PCA through O-demethylation (e.g., by VanAB or the tetrahydrofolate-dependent LigM) and to catechol via guaiacol O-demethylation, making G-rich softwoods—and the G fraction of any feedstock—highly compatible with PCA/catechol production [14]. By contrast, S-derived syringate requires a dedicated, often tetrahydrofolate-dependent O-demethylation system (e.g., DesA together with LigM in *Sphingobium* sp. SYK-6) to reach gallate/PCA—a capability absent from many candidate hosts—so that S-rich hardwoods are intrinsically more recalcitrant to funneling unless the relevant demethylase enzymes are introduced [14]. Conversely, the hydroxycinnamates abundant in grasses (*p*-coumarate, ferulate) and H-derived p-hydroxybenzoate are among the most readily assimilated substrates, entering the funnel toward PCA via well-characterized CoA-dependent and non-β-oxidative routes and via PobA-mediated hydroxylation.

This feedstock dependence has been demonstrated experimentally. Engineered *Sphingobium* SYK-6 derivatives funneling pulping black liquor to the downstream building block PDC achieved very high molar yields from G-rich softwood (Japanese cedar) liquor, whereas efficient conversion of S-rich hardwood (birch) liquor required additional pathway engineering to redirect syringyl-derived flux. A complementary opportunity is presented by C-lignin—a homopolymer of caffeyl alcohol found in certain seed coats—which can be depolymerized to catechol with minimal demethylation, representing an unusually direct feedstock for catechol production [10]. While significant progress has been achieved in elucidating microbial pathways for PCA and catechol production, it is important to distinguish between studies employing authentic lignin feedstocks and those using purified lignin-derived model compounds. Many of the metabolic engineering strategies discussed in subsequent sections have been demonstrated using individual aromatic substrates such as ferulic acid, vanillin, vanillate, syringate, p-hydroxybenzoate, or PCA rather than directly from native or technical lignin streams. Although these model compounds provide valuable platforms for pathway characterization and strain engineering, they do not fully capture the compositional heterogeneity, inhibitor profiles, and process-related challenges associated with real lignin feedstocks. Consequently, production metrics obtained with model substrates should not be interpreted as equivalent to direct lignin valorization, and translating these advances to complex lignin streams remains a critical challenge for industrial implementation.

## 4. Biofunneling Toward PCA: Converging Aromatic Streams into a Central Metabolite

Lignin depolymerization, accomplished through both enzymatic and non-enzymatic processes, generates a diverse range of aromatic monomers (e.g., ferulic acid, vanillin, vanillic acid, syringaldehyde, guaiacol, p-hydroxybenzoic acid), along with various dimers and other low-molecular-weight fragments [14,85,86]. Although numerous microorganisms exist in nature, only a limited number possess the metabolic capacity to convert these lignin-derived aromatics into PCA via pathways such as CoA-dependent β-oxidation, CoA-dependent non-β-oxidation, non-oxidative decarboxylation, and side-chain reductions. These bioconversion processes ultimately funnel aromatics into key metabolites, including vanillic acid, PCA, and catechol [14]. Subsequently, these intermediates are metabolized into acetyl-CoA, succinyl-CoA, or pyruvate, which enter the tricarboxylic acid (TCA) cycle to support cellular energy generation through aromatic ring-opening pathways such as the 2,3-cleavage pathway, 3,4-cleavage pathway and 4,5-cleavage pathway for PCA and the ortho-cleavage pathway for catechol [87]. By strategically modifying these metabolic pathways through genetic engineering and biofunneling strategies, the carbon flux can be redirected to enhance the microbial production of value-added aromatics such as PCA and catechol.

A major portion of lignin-derived aromatics originates from G-lignin units, primarily yielding ferulic acid and guaiacol. Microorganisms metabolize ferulic acid through several distinct but convergent pathways that ultimately produce vanillate. In the CoA-dependent β-oxidation pathway, ferulic acid is converted to feruloyl-CoA by feruloyl-CoA synthetase and sequentially processed by hydratases, dehydrogenases, and thiolases to generate vanillin and subsequently vanillic acid pathway [12,88]. A related CoA-dependent non-β-oxidative route converts feruloyl-CoA to vanillin via enoyl-CoA hydratase/lyase before its oxidation to vanillate by vanillin dehydrogenase [89]. Alternative pathways involve non-oxidative decarboxylation of ferulic acid to 4-vinylguaiacol, which is then oxidized to vanillin, or side-chain reduction to dihydroferulic acid followed by conversion into vanillic acid [4,90,91]. Regardless of the route, vanillate serves as the principal intermediate and is further demethylated by vanillate O-demethylase to produce PCA. From an engineering perspective, strengthening ferulate-to-vanillate conversion and enhancing O-demethylation efficiency, while simultaneously eliminating PCA-consuming enzymes, are key strategies for increasing PCA yields.

A second major class of lignin aromatics arises from H-lignin units, yielding *p*-coumarate, which is funneled to PCA through analogous peripheral routes. pCA is converted to pHBA through CoA-dependent β-oxidation processes involving activation by p-hydroxycinnamoyl-CoA synthetase, hydration and oxidation by enoyl-CoA hydratase, and cleavage by thiolases [92]. In an alternative CoA-dependent non-β-oxidation pathway, pHCHL converts p-hydroxycinnamoyl-CoA to 4-hydroxybenzaldehyde, which is then oxidized to pHBA [93]. Some bacteria utilize a non-oxidative decarboxylation pathway in which p-hydroxycinnamic acid decarboxylase generates p-hydroxyphenylpropionic intermediates that are subsequently transformed into pHBA. pHBA is then hydroxylated by p-hydroxybenzoate-3-hydroxylase to yield PCA [94]. Because H-lignin–derived intermediates converge predictably on pHBA and PCA, overexpressing pHCS, pHCHL, and pHB3H, coupled with suppression of downstream PCA degradation, represents a reliable strategy for enhancing PCA production from pCA-rich biomass streams.

**Figure 2:**
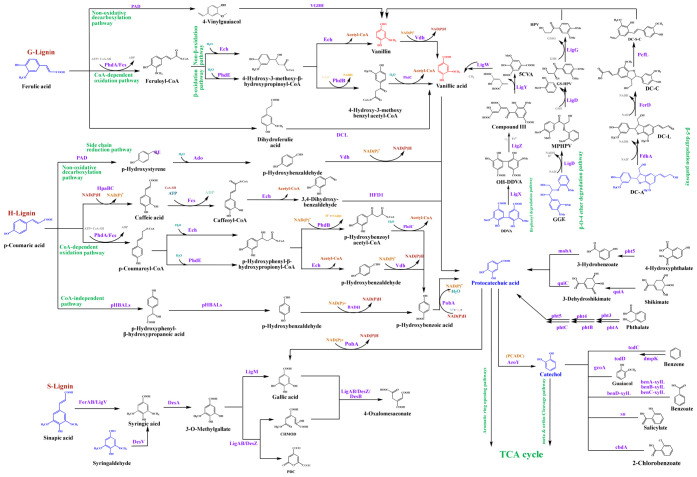
Comprehensive schematic of microbial biofunneling pathways converting lignin-derived aromatic compounds into PCA, catechol, and gallic acid. Lignin-derived substrates such as ferulic acid, p-coumaric acid, vanillin, syringic acid, benzoate, and phenol are metabolized via CoA-dependent β-oxidation, non-β-oxidative pathways, decarboxylation, and hydroxylation reactions. These pathways converge into key intermediates through enzymatic steps such as O-demethylation, dehydrogenation, and side-chain modification. The figure was adapted from previous studies [1,12,98]. Illustrations were created using ChemDraw Professional 22.2.0.3300.

In contrast, S-lignin–derived aromatics such as syringic acid are more recalcitrant due to the presence of two methoxy groups, which restrict enzymatic accessibility. Only a few specialized bacteria possess the tetrahydrofolate-dependent O-demethylases required to convert syringic acid into simpler intermediates [95]. In *Sphingomonas* sp. SYK-6, syringic acid is transformed into 3-O-methylgallic acid and then into gallic acid, which subsequently undergoes ortho- or meta-cleavage [96]. Natural pathways for direct conversion of syringic acid to PCA or catechol are uncommon, prompting metabolic engineering approaches that introduce heterologous demethylases, hydroxylases, and aromatic-converting enzymes into robust hosts such as *Pseudomonas putida*. Recent work demonstrates that syringic acid and other S-lignin aromatics can be redirected toward *cis,cis*-muconic acid, catabolic intermediate of catechol, through synthetic pathway reconstruction [97] (Figure 2).

**Figure 3:**
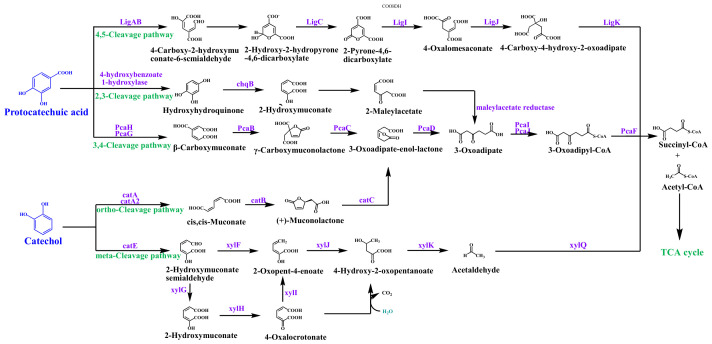
Comprehensive schematic of the ring-cleavage pathways of PCA and catechol. The figure was adapted from previous studies [1,12]. Illustrations were created using ChemDraw Professional 22.2.0.3300.

Across all three lignin units, bacterial metabolism reveals a strong convergence toward PCA as a central metabolic hub. G-lignin intermediates funnel through vanillate, H-lignin intermediates through pHBA, and both ultimately enter PCA-forming reactions catalyzed by demethylases or monooxygenases. This convergence provides a unique engineering advantage: modulating the activity of a small number of key enzymes can redirect the metabolism of a broad range of lignin-derived aromatics [99]. Blocking PCA ring-cleavage pathways (e.g., *pcaHG* or *ligAB* deletions) enables PCA accumulation, while introducing PCADC and inhibiting catechol-cleaving dioxygenases allows efficient catechol production from lignin substrates (Figure 3).

Collectively, these pathways highlight multiple promising engineering opportunities for producing PCA and PCA-derived platform chemicals. Enhancing upper-pathway flux through overexpression of FCS, ECH, VDH, pHCS, pHB3H, and *vanAB*; eliminating native ring-cleavage reactions; improving aromatic uptake through transporter engineering; and introducing heterologous catalytic modules such as LigAB/LigI variants or evolved O-demethylases can dramatically increase PCA titers [14,65]. Furthermore, rewiring PCA metabolism enables production of a broad portfolio of high-value derivatives, such as 2-pyrone-4,6-dicarboxylic acid (PDC), β-ketoadipate (β-KA), *cis,cis*-muconic acid (cc-MA), and catechol, positioning PCA as a versatile metabolic hub for lignin valorization [14]. Integrating these strategies with CRISPR-enabled regulation, dynamic control circuits, and tolerance engineering will be essential for developing next-generation microbial cell factories capable of efficiently upgrading lignin into PCA and its downstream economically viable aromatic biochemicals.

## 5. Engineering Catechol Node: Native Routes and Synthetic Diversions

Several aromatic chemicals, including benzoate, toluene, cinnamic acid, and phenolics, are produced when lignin depolymerizes. Although they can be converted to catechol via several enzymatic pathways [1], their formation is less than that of other key lignin aromatics. Most bacteria can break down lignin aromatics through different enzymatic cascades, with PCA being an intermediate product. However, some bacteria possess unique enzymatic systems that bypass PCA and directly convert lignin aromatics into catechol. Metabolic engineering can further broaden this capability by redirecting flux from PCA into catechol, offering an alternative route to catechol-derived value-added chemicals (Figure 2).

In the canonical upper lignin degradation route, G-lignin–derived compounds (e.g., ferulic acid, β-aryl ethers, biphenyls) are metabolized to vanillin and then oxidized to vanillic acid, which is subsequently demethylated by vanillate O-demethylase to yield PCA. However, certain bacteria utilize a non-oxidative decarboxylation of vanillic acid via vanillate decarboxylase (VDC) to produce guaiacol directly—circumventing PCA formation. For example, in strains such as *Bacillus megaterium* and *Streptomyces* sp., [100] or vanillic acid undergoes non-oxidative decarboxylation to guaiacol, which is then demethylated by a P450 aromatic O-demethylase to generate catechol [101]. In the case of *Amycolatopsis* sp. ATCC 39116, this is enabled by a streamlined electron-transfer system in which a single redox partner, GcoB, delivers electrons from NADH to the P450 enzyme GcoA, enabling efficient guaiacol demethylation [102].

Lignin depolymerization can also yield simpler aromatics such as benzoic acid and phenols via oxidative cleavage reactions (e.g., catalyzed by DyP-type peroxidases) [103]. These small aromatic acids and phenols may be funneled into catechol through classical aromatic degradation pathways. For instance, benzoate can be converted by benzoate 1,2-dioxygenase to cis-1,2-dihydroxycyclohexane-3,4-diene-1-carboxylate, which after dehydrogenation yields catechol [104]. Similarly, phenol may be converted directly to catechol by phenol hydroxylase (vanillin synthase/VanS in some reports), while salicylic acid (when present) may be hydroxylated by salicylate 1-hydroxylase to catechol [105]. In particular, soil-dwelling bacteria such as *Rhodococcus opacus* PD630 have demonstrated the ability to degrade complex phenolic mixtures from lignin depolymerization and convert phenol into catechol via such enzymatic systems [105].

In practice, most efficient lignin-to-catechol processes combine two strategies: (1) leveraging natural catechol-forming pathways (e.g., via VDC or phenol hydroxylase) when aromatics like vanillate, guaiacol, benzoate, or phenol are abundant; or (2) engineering bacteria to redirect PCA toward catechol, especially if lignin-derived feedstocks are enriched in G-lignin or H-lignin units that preferentially yield vanillate or p-hydroxybenzoate (pHBA) [14]. Because H- and G-lignin–derived fragments tend to funnel predictably into vanillate or pHBA—and then PCA—these feedstocks are particularly amenable to metabolic rerouting strategies. Conversely, lignin breakdown routes that yield substantial benzoate or phenol may be ideal for exploiting native catechol-producing pathways [103]. In either case, combining pathway engineering (e.g., overexpressing key upstream enzymes, integrating VDC or phenol hydroxylases, deleting competing ring-cleavage dioxygenases) with improved substrate uptake and detoxification can significantly boost catechol yield and expand the range of usable lignin feedstocks [106].

## 6. Metabolic Entry Points: Controlling Carbon Flux at Aromatic Junctions

Lignin depolymerization generates aromatic acids, aldehydes, and phenols that differ in side-chain length, oxidation state, and ring substitution. Before entering PCA- or catechol-centered pathways, these compounds undergo preparatory reactions such as side-chain oxidation or shortening, hydroxylation, and O-demethylation (Figure 2) [106]. These reactions do not constitute ring cleavage but instead standardize chemically heterogeneous substrates into dihydroxylated aromatic structures compatible with downstream dioxygenases. PCA formation occurs predominantly from methoxylated lignin-derived aromatics following O-demethylation reactions that yield a 3,4-dihydroxybenzoate structure [14]. This transformation commits aromatic carbon to PCA-dependent ring-cleavage pathways, including the 3,4-, 4,5-, or 2,3-cleavage routes. In contrast, catechol arises mainly from non-methoxylated or decarboxylated aromatics through hydroxylation or dioxygenation reactions that generate a 1,2-dihydroxybenzene structure [107]. These structural differences define the enzymatic compatibility of substrates with specific cleavage systems and explain the coexistence of PCA- and catechol-based pathways in lignin-degrading microorganisms.

Once formed, PCA and catechol occupy a critical metabolic position. In native systems, both intermediates are rapidly cleaved by dioxygenases and funneled into central metabolites such as acetyl-CoA, succinyl-CoA, or pyruvate, which enter the TCA cycle. Because PCA and catechol are redox-active and potentially toxic, microorganisms tightly regulate their formation and rapidly channel them into downstream ring-cleavage pathways [108]. Consequently, the cellular concentration of PCA and catechol is typically low and transient under physiological conditions. From a biotechnological perspective, the entry of lignin-derived intermediates into PCA or catechol pathways represents a decisive control point. Inhibition or deletion of downstream ring-cleavage enzymes enables accumulation of PCA or catechol [108]. Because diverse lignin-derived aromatics converge on a small number of such entry reactions, manipulating these steps provides an efficient strategy for redirecting carbon flux without extensive pathway reconstruction [14]. From a metabolic engineering standpoint, controlling pathway entry and blocking ring-cleavage reactions enables stabilization and accumulation of these transient intermediates.

## 7. PCADC as a Synthetic Bridge: Linking PCA Accumulation to Catechol Production

### 7.1. Enzymatic Mechanism, Accessory Proteins, and Host Compatibility

Enzyme PCADC plays a pivotal role in enabling the conversion of PCA to catechol, thereby linking PCA-centered aromatic metabolism to catechol-based degradation and biosynthesis pathways [46]. Although this reaction is not part of the canonical β-ketoadipate pathway, PCADC has emerged as a key enzymatic tool in metabolic engineering strategies aimed at redirecting lignin-derived carbon flux toward catechol and its downstream value-added products. PCADC catalyzes the non-oxidative decarboxylation of PCA to catechol with the release of CO_2_. This reaction belongs to the class of lyase-mediated decarboxylation reactions and does not require external cofactors such as NAD(P)H or ATP [38]. The enzyme exploits the dihydroxylated aromatic ring of PCA to stabilize a carbanion intermediate during decarboxylation, making the reaction thermodynamically feasible under physiological conditions. Structural and mutational analyses indicate that hydrogen bonding and metal-independent active-site residues are critical for orienting PCA and facilitating CO_2_ release [38,106].

PCADC belongs to the UbiD family of reversible aromatic decarboxylases and operates via a prenylated-FMN (prFMN) cofactor rather than the cofactor-free chemistry assumed earlier [109,110]. AroY, the canonical PCADC of *Klebsiella pneumoniae* A170-10 and *Enterobacter cloacae*, shares 35–45% sequence identity with archetypal UbiD enzymes and houses prFMN in a central β-barrel domain [38,106,110]. The cofactor is built in two steps from FMN and dimethylallyl monophosphate by a UbiX-family flavin prenyltransferase—KpdB in the *K. pneumoniae* system [106] and EcdB in the *E. cloacae* system [38]; without it *AroY* is catalytically silent. A second class of accessory factor (KpdC/KpdD in *K. pneumoniae*, EcdD in *E. cloacae*) acts as molecular chaperones for *prFMN* insertion and holoenzyme assembly [106,111]. The practical consequence is that any heterologous PCADC implementation requires co-expression of at least two accessory genes alongside *aroY*; minimal constructs that omit them—common in early literature—typically produce <10% of the activity obtained with the full system [38]. Introduction of *kpdB* alone raised catechol yield in *P. putida* KT2440 to 98.5% from mixed lignin-derived aromatics, confirming that prFMN supply, not AroY abundance, is the dominant in vivo bottleneck.

In nature, PCADC activity is relatively rare and is not widely distributed among lignin-degrading bacteria. Most microorganisms metabolize PCA exclusively through ring-cleavage pathways (e.g., PCA 3,4-dioxygenase or PCA 4,5-dioxygenase), leading to complete aromatic mineralization rather than catechol formation [53,112]. Consequently, direct biological conversion of PCA to catechol is uncommon in wild-type strains, and catechol biosynthesis typically proceeds from non-methoxylated aromatics such as phenol, benzoate, or salicylate [113,114]. The limited natural role of PCADC underscores its importance as a synthetic metabolic bridge rather than a dominant physiological enzyme. Its introduction into engineered hosts enables bypassing native PCA ring-cleavage routes and establishes a direct biochemical link between PCA accumulation and catechol production.

**Figure 4:**
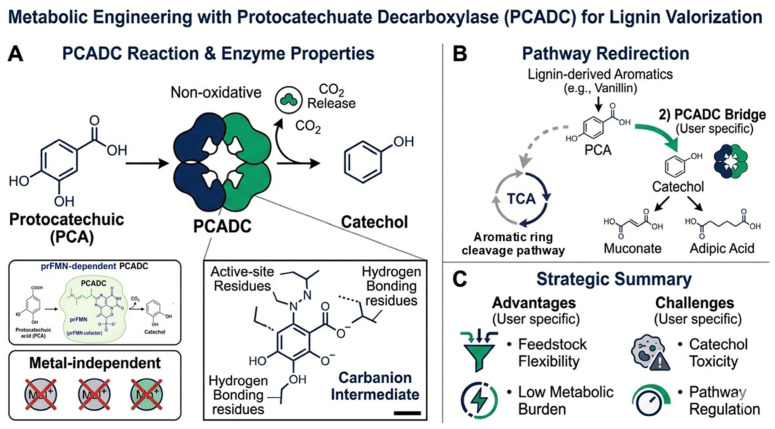
PCADC-mediated redirection of lignin-derived aromatics to catechol. (**A**) PCADC catalyzes the non-oxidative decarboxylation of protocatechuic acid (PCA) to catechol with CO_2_ release via a carbanion intermediate; the reaction is cofactor- and metal-independent. (**B**) PCADC introduces a synthetic bypass that redirects PCA from native ring-cleavage pathways toward catechol and downstream products such as *cis,cis*-muconic acid and adipic acid. (**C**) Advantages include feedstock flexibility, low metabolic burden, and oxygen-insensitivity—unlike many ring-cleaving dioxygenases, PCADC is cofactor- and O_2_-independent, making it well suited to large-scale fermentation under microaerobic or anaerobic conditions—whereas challenges involve catechol toxicity and pathway regulation.

PCADC is strictly specific for para-hydroxylated aromatic acids bearing an ortho-hydroxyl: PCA and gallate are decarboxylated efficiently, whereas vanillate, syringate and ferulate are essentially inert until prior O-demethylation regenerates the catechol moiety. Purified *AroY* holoenzyme shows kcat in the 1–10 s^−1^ range with KM for PCA of 0.2–2 mM [38,106]—one to two orders of magnitude below the ring-cleavage dioxygenases (*PcaHG, CatA*) competing for the same PCA pool, which is why PCADC expression must always be paired with *pcaHG* deletion. Two further limitations have direct process implications: product inhibition by catechol above ~5–10 mM (creating a self-limiting feedback that intersects with the catechol toxicity, and oxygen sensitivity of the prFMN cofactor itself [109,110]—so PCADC-dependent processes commonly benefit from microaerobic or anaerobic operation (Figure 4) [38].

From a pathway-design perspective, PCADC offers several advantages. First, it enables catechol biosynthesis from methoxylated lignin aromatics, which otherwise favour PCA accumulation. Second, because the reaction is non-oxidative and cofactor-independent, PCADC imposes minimal metabolic burden on the host. Third, catechol produced via PCADC can be readily channelled into downstream pathways for the synthesis of *cis,ci*-muconic acid, adipic acid, or polymer precursors, expanding the economic potential of lignin valorization [49,51,79]. However, catechol toxicity remains a significant challenge. Consequently, PCADC-based strategies are most effective when coupled with tight regulation of catechol dioxygenases, dynamic pathway control, or rapid conversion of catechol into downstream products to prevent intracellular accumulation and oxidative stress [38,55].

Industrial-host compatibility hinges on three properties: a native UbiX-family prenyltransferase, sufficient cytoplasmic FMN and DMAP pools, and microaerobic tolerance. *P. putida* KT2440 satisfies all three—it carries a native UbiX-like activity, its solvent-tolerant membrane chemistry supports microaerobic operation, and central metabolism delivers ample FMN—making it the host of choice for lignin-to-catechol processes [15]. *E. coli* requires co-expression of KpdB or EcdB because its native UbiX is dedicated to ubiquinone biosynthesis, yet strains carrying the full AroY/KpdBCD cassette achieve gram-scale catechol from shikimate-derived PCA [115]. *C. glutamicum* lacks a fully characterised UbiX system and shows lower PCADC activity even with accessory co-expression [111]. In any non-*Pseudomonas* chassis, PCADC genes should be expressed under tunable rather than constitutive promoters to balance accessory supply against AroY load, and prFMN biosynthesis precursors (FMN, DMAP) should be reinforced—these molecular interventions plus process-level oxygen control are what separate recent gram-per-litre titres from the milligram-scale yields of early proof-of-concept studies [15,46].

### 7.2. Critical Perspective: Remaining Challenges for PCADC-Driven Catechol Production

Despite remarkable advances in PCADC engineering, current studies collectively indicate that efficient catechol production depends less on the introduction of a single heterologous enzyme than on coordinated optimization of the entire aromatic conversion network [15,38,106,116]. Comparison of reported engineering strategies suggests that *Pseudomonas putida* KT2440 currently represents the most promising microbial chassis for lignin-to-catechol bioconversion because of its intrinsic aromatic tolerance, broad substrate utilization, solvent-resistant membrane, and well-characterized β-ketoadipate pathway [15,46,117,118]. These physiological advantages enable efficient utilization of heterogeneous lignin-derived aromatics while maintaining redox balance during aromatic metabolism [116,119]. In contrast, *Escherichia coli* requires extensive pathway reconstruction because it lacks native lignin-catabolic pathways [115,120], whereas *Corynebacterium glutamicum* remains attractive for industrial fermentation but currently exhibits lower PCADC activity and less efficient aromatic assimilation than *P. putida* [121].

Comparison of recent metabolic engineering studies further reveals that PCADC itself is no longer the only rate-limiting step. Although co-expression of AroY with the KpdBCD or EcdBD accessory systems substantially improves PCA decarboxylation [38,106], efficient catechol accumulation additionally requires elimination of competing ring-cleavage pathways (*pcaHG*, *catA*), enhancement of upstream aromatic funneling enzymes such as *VanAB* and *PobA*, and reinforcement of prFMN cofactor biosynthesis [15,46]. Multi-omics and ^13^C-flux analyses consistently identify aromatic O-demethylation, prFMN availability, and metabolic flux partitioning as the principal bottlenecks limiting carbon conversion from lignin-derived aromatics to catechol [116].

Although several engineered strains report catechol yields approaching complete conversion from individual aromatic substrates [15,46,106], these values should be interpreted cautiously from an industrial perspective. Most reported yields were obtained using purified lignin-derived model compounds, including ferulate, vanillate, *p*-coumarate, or PCA, under controlled laboratory conditions rather than authentic lignin hydrolysates [46]. Real lignin streams generated by kraft pulping, alkaline pretreatment, or organosolv fractionation contain complex mixtures of aromatic monomers, oligomers, residual carbohydrates, organic acids, inorganic salts, and fermentation inhibitors whose composition varies with biomass source and pretreatment chemistry [84,100,116,122,123]. Consequently, substrate heterogeneity frequently reduces aromatic uptake, perturbs metabolic flux distribution, and lowers product yields compared with model substrate studies [116,119,124].

Scalability is additionally constrained by catechol toxicity. Intracellular catechol readily undergoes auto-oxidation to reactive quinones, generating oxidative stress, damaging cellular macromolecules, and creating product inhibition that ultimately limits fermentation performance [125]. Therefore, high laboratory conversion efficiencies do not necessarily translate into economically viable production unless catechol synthesis is tightly coupled with downstream conversion [48,49], in situ product removal [126], transporter engineering [127,128], or adaptive laboratory evolution to enhance aromatic tolerance.

Overall, recent studies suggest that future progress will depend less on discovering new PCADC enzymes than on integrating feedstock-specific biofunneling [116,119], balanced cofactor engineering [15,38,106], dynamic metabolic regulation [31,32], tolerance engineering [129,130], and downstream process intensification [48,49]. Such integrated strategies will be essential for translating the high conversion efficiencies observed with model aromatic substrates into robust industrial bioprocesses capable of valorizing authentic lignin streams.

Comparison of the major microbial chassis used for PCA and catechol production indicates that no single host is optimal for every lignin feedstock, and host selection should be guided by the desired substrate spectrum and process requirements (Table 1). *Pseudomonas putida* KT2440 currently represents the most mature chassis for lignin valorization because it combines a native β-ketoadipate pathway [117], broad utilization of H- and G-type lignin aromatics [15,118], endogenous prFMN biosynthesis through a UbiX-like system [15], high solvent and aromatic tolerance [130,131], and an extensive genetic engineering toolbox [55]. These characteristics collectively explain why *P. putida* consistently delivers the highest catechol yields from lignin-derived aromatics [15,46]. In contrast, *Corynebacterium glutamicum* offers exceptional industrial robustness and tolerance toward aromatic compounds, making it an attractive future production host [121]; however, its limited native aromatic metabolism and incomplete prFMN/PCADC system currently necessitate substantial metabolic reconstruction [121]. *Escherichia coli* remains the most genetically tractable chassis and is widely used for proof-of-concept pathway engineering [115,120], yet its lack of native lignin-catabolic pathways, dependence on heterologous PCADC accessory proteins, and poor catechol tolerance considerably restrict its industrial applicability [115]. Meanwhile, *Sphingobium* sp. SYK-6 possesses an unparalleled capacity to metabolize syringyl-rich (S-type) lignin because of its specialized aromatic catabolic network, but comparatively limited genetic tools and lower process robustness reduce its suitability as a large-scale production host [132]. Overall, these comparisons suggest that *P. putida* is presently the leading chassis for direct conversion of heterogeneous lignin streams [46,121], whereas *C. glutamicum* may emerge as the preferred industrial host once efficient prFMN biosynthesis and PCADC functionality are established. Future strain engineering should therefore prioritize host-specific optimization rather than adopting universal engineering strategies.

## 8. Rewiring Microbial Metabolism for Targeted PCA Accumulation

**Figure 5:**
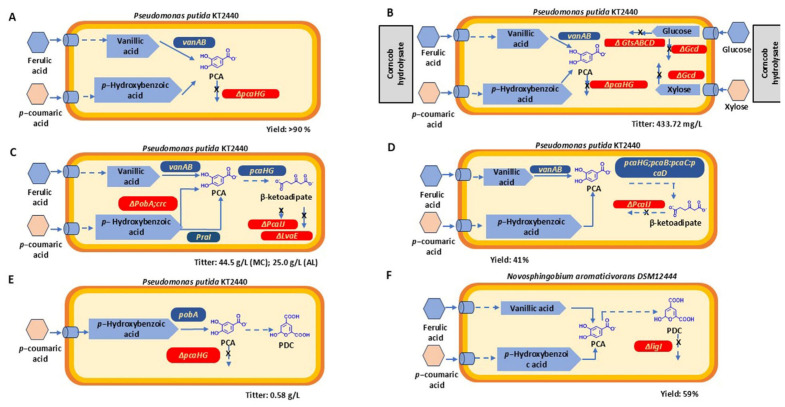
Engineering strategies for PCA production and production of other compounds through PCA. Engineering strategies for PCA (**A**,**B**) [22,46], β-ketoadipate (**C**,**D**) [124,138], PDC (**E**,**F**) [139,140] from lignin aromatics using different bacterial cell factories. PCA: protocatechuate; PDC: 2-Pyrone-4,6-dicarboxylic acid. The genes in a blue box represent those cloned or overexpressed in the host system. The genes in a red box represent those deleted from the host system.

Modifying the host bacteria’s metabolic system could produce PCA and other valuable compounds from lignin aromatic compounds through PCA. Beyond lignin substrates, PCA has also been produced from methanol in *Corynebacterium glutamicum*, highlighting the broader substrate versatility [137]. The major metabolic engineering strategies for PCA production from lignin reported previously were listed in Table 2 and Figure 5. The 3,4-cleavage pathway is more frequent in most bacteria [22,119], and blocking this pathway resulted in PCA accumulation in the bacterial cell factories. This pathway starts with an enzyme called protocatechuate 3,4-dioxygenase encoded by the *pcaHG* gene; deleting this gene or introducing mutations that render the enzyme non-functional, the conversion of PCA to downstream metabolites can be prevented or significantly reduced. This alteration in the metabolic pathway directs the flux towards PCA accumulation rather than its further degradation. The *pcaHG* knockout mutants will retain PCA within its metabolic pathway, allowing it to accumulate as the primary end product [21,22]. The *P. putida* KT2440 strain has been engineered to produce PCA from corncob hydrolysate. The *pcaHG* gene has been deleted from the pathway by homologous recombination knockout technology using a pEASY-Blunt cloning vector, while the *vanAB* gene encoding the V-O-DML has been overexpressed to enhance the conversion of vanillic acid to PCA. This modified system yields 433.72 mg/L of PCA from corncob hydrolysate [22]. The *pcaHG* and *catA/B* genes have been deleted from the KT2440 strain’s metabolic system while overexpressing the *vanAB; PobA* genes encode the vanillate O-demethylase and p-hydroxybenzoate hydroxylase, respectively, to produce PCA from lignin aromatics [119].

Beyond PCA accumulation, strategic modulation of downstream PCA cleavage pathways has enabled the biosynthesis of additional high-value intermediates, such as β-ketoadipate (β-KAP). β-KAP is a widely recognized precursor compound used to produce biodiesel and pesticides. β-KAP acid is a major intermediate in the protocatechuate 3,4-dioxygenase ring cleavage pathway. Production of β-KAP from lignin by bacteria could be a sustainable approach; however, it requires genetic engineering to make the bacteria accumulate β-KAP. Scientists recently engineered a model strain of *P. putida* KT2440 to efficiently convert lignin aromatics into β-KAP. The global regulator *crc* gene, which likely reduces competition for precursor molecules needed for β-KAP production, has been deleted from the system while overexpressing genes encoding enzymes responsible for pHB3H and V-O-DML. This modified system produced β-KAP from lignin aromatics and corn stover-derived lignin aromatics at about 1.15 g/L and 0.66 g/L, respectively [124]. Most genetic engineering approaches were adopted in the PCA branches of the β-KAP pathway to produce the β-KAP as an end product. A recent study by Suzuki et al. modified the 4-dioxygenase ring cleavage pathway by overexpressing the key genes *vanAB*:*pcaHG*:*pcaB*:*pcaC*:*pcaD* to produce β-KAP from vanillin as a substrate. This system produces 23 g/L of β-KAP from 25 g/L of vanillin with a 93% conversion rate [135].

In parallel to β-KAP biosynthesis, alternative PCA cleavage routes have been exploited for the production of 2-pyrone-4,6-dicarboxylic acid (PDC). PDC is a promising compound for polyester production, naturally occurring as a metabolite during lignin degradation by microbes like *Sphingobium* sp. SYK-6. As interest in addressing plastic-related issues grows, PDC’s significance as a raw material also rises, with the potential to replace terephthalic acid in polyester manufacturing. Various PDC-based polyesters have been reported to exhibit impressive functional properties [127]. Production of PDC from the lignin aromatics materials could be challenging; it requires pathway engineering in host bacteria. In this, the most thoroughly studied bacterium for enzymatic and genetic analysis of lignin-derived aromatic compounds via the PCA 4,5-cleavage pathway is *Sphingobium* sp. SYK-6. [132]. The aromatic degrading *N. aromaticivorans* DSM12444 uses the 4,5-cleavage pathway to degrade the PCA. Recently, this strain has been engineered to produce the PDC from ferulic acid and pCA; the *ligI* gene, which hydrolyzes PDC, has been deleted [140].

**Table 2:** Engineering Strategies for PCA production and other compounds production through PCA (for recent 10 years).

Strain	Feedstock Type *	Substrate	Engineering Strategy	Product	Titter	Yield/Conversion	Productivity	Scale	Main Limitation	Reference
*P. putida* KT2440	Mixed (MC + AL)	Model lignin monomers; biomass hydrolysates	Δ*pcaHG*; *vanAB*	PCA	Not reported	>90% (p-CA); >50% (vanillate)	NR	Flask	Yield reported, titer unavailable	[21]
*P. putida* KT2440	MC	Ferulic acid + p-CA	Δ*pcaHG*; *vanAB*, *HcnK*, *PobA*	PCA	12.7 g/L	Not reported	NR	Flask	Model compounds only	[117]
*P. putida* KT2440	Mixed (MC + AL)	Pure p-CA; corncob hydrolysates	Δ*pcaHG*; *vanAB*	PCA	433.7 mg/L (hydrolysate 2); 253.9 mg/L (hydrolysate 1)	97.7% (pure p-CA); 56.7–70.9% (hydrolysates)	NR	Flask	Significant reduction with real hydrolysates	[22]
*P. putida* KT2440	MC	p-CA/mixed aromatics	Δ*pcaHG*; *pobA*, *vanAB*	PCA	17.5 g/L	94.5%	NR	Fed-batch	Model aromatic mixture	[118]
*P. putida* KT2440	MC	Ferulic acid + p-CA	Δ*pcaHG*; *ligABC*	PDC	22.7 g/L	100% mol/mol	0.21 g/L/h	Fed-batch	Demonstrated only with model compounds	[141]
*P. putida* KT2440	MC	p-CA	Δ*pcaHG*; *pobA*, *ligAB*	PDC	0.58 g/L	52%	NR	Resting-cell	Low titer; non-growing process	[139]
*N. aromaticivorans DSM12444*	Mixed (MC + AL)	Vanillate + p-CA; poplar lignin depolymerization liquor	Δ*ligI*	PDC	Not reported	59% from lignin liquor; 22–100% from model compounds	NR	Flask	Feedstock-dependent performance	[140]
*P. putida PpY1100*	Mixed (MC + AL)	Vanillin; lignosulfonate extracts	*vanAB*, *ligABC*	PDC	Not reported	~100% conversion	NR	Flask	Titer not reported	[142]
*P. putida PpY1100*	MC	Vanillic acid	*vanAB*, *ligABC*	PDC	99.9 g/L	~100% conversion	Not reported	Optimized fed-batch	Achieved using pure substrate only	[52]
*P. putida* KT2440	Mixed (MC + AL)	Model LRCs; corn stover-derived LRCs	Δ*crc*, *pcaIJ*, *LvaE*; *vanAB*, *pcaHG*, *PraI*	β-KAP	44.5 g/L (MC); 25.0 g/L (AL)	100% (MC)	1.15 g/L/h (MC); 0.66 g/L/h (AL)	Fed-batch	Reduced productivity with lignin-derived feedstock	[124]
*P. putida* KT2440	MC	Vanillin/vanillate	*vanAB*, *pcaHG*, *pcaBCD*	β-KAP	~23 g/L	≥93%	NR	1-L culture	Pure substrate process	[135]
*P. putida* KT2440	MC	Ferulic acid + p-CA (via PCA)	Δ*pcaJ*; *vanAB*, *pcaHG*, *pcaBCD*	β-KAP	Not reported	41%	NR	Flask	Carbon loss during downstream processing	[138]
*P. putida* KT2440	MC	PCA	Δ*pcaD*; *vanAB*, *pcaHG*, *pcaBC*	KEL (muconolactone)	Not reported	Not reported	NR	Flask	Product accumulation demonstrated; quantitative data unavailable	[138]

* Feedstock Type: MC = lignin-derived model compound; AL = authentic lignin stream (lignin hydrolysate, black liquor, alkaline pretreatment liquor, kraft lignin fraction, etc.); NR: Not Reported.

Overall, engineering the PCA node represents a central and versatile strategy for lignin valorization, enabling not only efficient PCA accumulation but also controlled diversion toward downstream products such as β-ketoadipate and PDC. Selective blocking of PCA ring-cleavage pathways, coupled with reinforcement of upstream funneling reactions, has proven effective across multiple microbial hosts. Future work should focus on systems-level optimization, including dynamic regulation of PCA flux, alleviation of regulatory constraints, and improved tolerance to aromatic intermediates. Integrating these metabolic strategies with real lignin feedstocks and scalable bioprocess designs will be crucial for translating PCA-centered engineering into industrially viable platforms.

## 9. Redirecting Aromatic Flux: Advanced Strategies for Catechol Biosynthesis

**Figure 6:**
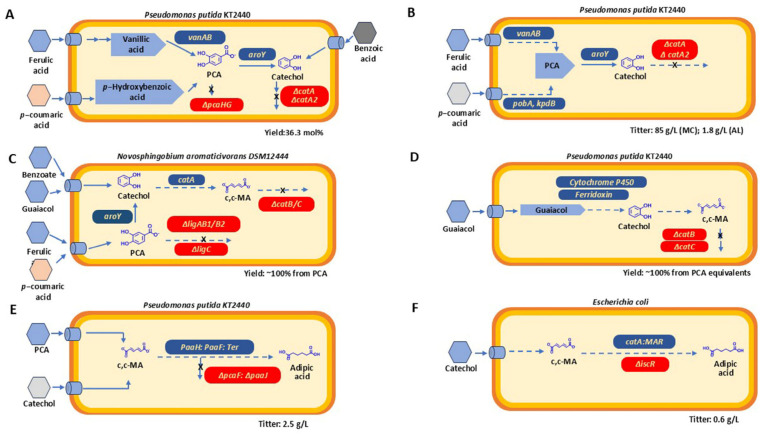
Engineering strategies for catechol production and other compounds production through catechol. Engineering strategies for catechol (**A,B**) [15,46], *cis,cis*-muconic acid (c,c-MA) (**C,D**) [50,122], adipic acid (**E**,**F**) [51,146] from lignin aromatics using different bacterial cell factories. c,c-MA: *cis,cis*-muconate. The genes in a blue box represent those cloned or overexpressed into the host system. The genes in a red box represent those deleted from the host system.

Metabolic engineering strategies for catechol production from lignin have primarily focused on preventing its further degradation by deleting genes encoding catechol ring-cleavage enzymes. In particular, enzymes initiating catechol ortho- and meta-cleavage are common targets for gene knockout, thereby enabling catechol accumulation [15]. Several studies have successfully redirected PCA metabolism toward catechol to generate valuable chemicals such as *cis,cis*-muconic acid [46,121]. In addition to pathway blocking, the source and routing of catechol precursors play a crucial role in determining production efficiency. Catechol formation from intermediates such as vanillic acid and benzoic acid is naturally observed in some bacterial systems [143] (Table 3; Figure 6). However, most lignin-based catechol production strategies rely on redirecting PCA catabolism via the heterologous expression of PCADC. To prevent downstream ring cleavage, this strategy is commonly coupled with deletion of *pcaHG* (encoding protocatechuate 3,4-dioxygenase) and *catA/A2* (encoding catechol 1,2-dioxygenase), thereby blocking both PCA and catechol ring cleavage [97].

Building on this concept of dual pathway control, recent studies have demonstrated the effectiveness of combining gene deletions with synthetic pathway insertion. Recent work has combined simultaneous deletion of PCA- and catechol-degrading genes with PCADC expression to enable efficient catechol biosynthesis [46]. In *Pseudomonas putida* KT2440, catechol production from ferulic acid and p-coumaric acid was achieved by expressing a codon-optimized *aroY* gene from *Enterobacter cloacae*, encoding PCADC, while enhancing vanillic acid conversion through overexpression of *vanAB* from *Acinetobacter* sp. ADP1. Concurrent deletion of *pcaHG* and *catA/A2* effectively redirected metabolic flux toward catechol accumulation [46]. Similarly, introduction of *aroY* together with accessory proteins EcdB and EcdD enabled efficient diversion of PCA catabolism toward catechol in *P. putida* KT2440 [38]. Recently, Zhou et al. further enhanced catechol biosynthesis in *P. putida* KT2440 by combining deletion of *pcaHG*, *catA*, and *catA2* with heterologous expression of *aroY* and overexpression of the rate-limiting enzymes *vanAB* and *pobA*. Additional cofactor engineering through *kpdB* expression improved decarboxylase activity, resulting in a catechol yield of 98.5% from mixed lignin-derived aromatics (ferulic acid and p-coumaric acid). This study highlights the importance of integrating pathway blocking, bottleneck removal, and cofactor optimization to maximize aromatic carbon flux toward catechol production [15].

These studies collectively demonstrate that PCADC is a key enabling enzyme for catechol production from H- and G-lignin aromatics via PCA. Recent multi-omics and 13C-fluxomics analyses of *P. putida* KT2440 identified *VanAB*, *PobA*, and *PcaHG* as key bottlenecks during lignin-derived aromatic utilization. The study further revealed that efficient aromatic conversion requires metabolic remodeling and cofactor balancing to maintain NAD(P)H and ATP homeostasis, providing valuable insights for future engineering of PCA- and catechol-producing strains [116]. Two well-characterized PCADC systems have been widely applied: (i) *aroY* with *EcdB/EcdD* from *Enterobacter cloacae* [38] and (ii) *aroY* with *KpdB/KpdC/KpdD* from *Klebsiella pneumoniae* subsp. *pneumoniae* A170-10 [106]. While effective, further optimization of PCADC activity and enzyme engineering remains essential for industrial-scale catechol production. Beyond lignin substrates, catechol has also been produced from glucose by rewiring the shikimate pathway in *Escherichia coli*, underlining the broader applicability of PCADC-based strategies [115]. Importantly, catechol serves as a direct precursor for several high-value downstream chemicals, most notably *cis,cis*-muconic acid (c,c-MA), a high-value platform chemical, primarily used as a precursor for adipic acid synthesis via hydrogenation [48,49]. In microbial systems, c,c-MA biosynthesis typically proceeds through catechol as a central intermediate, whereas most lignin-derived aromatics are initially funneled through PCA. Consequently, redirecting PCA catabolism toward catechol is a key requirement for c,c-MA production from lignin. This is commonly achieved by introducing PCADC while blocking competing PCA cleavage pathways. For example, *Novosphingobium aromaticivorans* DSM12444 was engineered by expressing PCADC and catechol 1,2-dioxygenase (*catA*) while disabling the PCA 4,5-cleavage pathway, resulting in complete conversion of alkaline pretreatment liquor into c,c-MA [122]. In *Sphingobium* sp. SYK-6, introduction of PCADC, together with the acetovanillone synthetase (*acvABCDEF*) gene cluster and deletion of *catB* and *pcaHG*, enabled c,c-MA production from acetovanillone via catechol [144]. Beyond the bacterial strains, the c,c-MA has been produced from lignocellulosic biomass by strategically engineering the *Saccharomyces cerevisiae* [145].

Beyond PCA-derived routes, alternative pathways exploiting benzoate metabolism have also been explored. In *P. putida* KT2440, deletion of *catB* and *catC* combined with expression of cytochrome P450 and ferredoxin enabled efficient conversion of guaiacol to c,c-MA via catechol, achieving complete guaiacol-to-catechol conversion [50]. Additional improvements include the removal of enzymes responsible for c,c-MA degradation and enhancement of catechol 1,2-dioxygenase activity [143]. More recently, *P. putida* KT2440 was engineered to convert multiple lignin types, including S-lignin, into c,c-MA through heterologous pathway integration [97]. Global regulatory systems also influence aromatic utilization. Carbon catabolite repression regulators such as Crc suppress lignin aromatic metabolism in the presence of preferred carbon sources [124]. Disruption of *crc* has therefore emerged as an effective strategy to enhance lignin-derived carbon flux toward target products.

Adipic acid, a major industrial chemical with a global demand exceeding 2.6 million tons annually, is traditionally produced from petrochemical feedstocks [147]. One biological strategy involves condensation of succinyl-CoA and acetyl-CoA to form 3-ketoadipoyl-CoA, which is subsequently converted to adipic acid via enzymatic reduction and dehydration steps [148,149,150]. Engineered *E. coli* strains have demonstrated high adipic acid titers, reaching 68.0 g/L with a 72.7% theoretical yield under fed-batch conditions [150].

Overall, catechol-centered metabolic engineering has emerged as a powerful framework for lignin valorization, enabling the conversion of heterogeneous lignin-derived aromatics into high-value products such as catechol, *cis,cis*-muconic acid, and adipic acid [136]. Strategic blockage of PCA and catechol ring-cleavage pathways, combined with the introduction of PCADC, has proven essential for effective flux redirection. Future research should emphasize systems-level optimization, including dynamic regulation, relief of global regulatory constraints (e.g., Crc), and enhancement of PCADC activity and tolerance to aromatic toxicity. Integrating these advances with real lignin feedstocks and robust industrial hosts will be critical for achieving scalable and economically viable bioprocesses.

**Table 3:** Engineering Strategies for catechol production and other compounds production through catechol (for recent 10 years).

Strain	Feedstock Type *	Substrate	Engineering Strategy	Product	Titter	Yield/Conversion	Productivity	Scale	Main Limitation	Reference
*P. putida* KT2440	MC	Ferulic acid, p-CA	Δ*catA/A2*, Δ*pcaHG*; *vanAB*, *aroY*	Catechol	NR	36.3 mol%	NR	Flask	Model compounds only	[46]
*P. putida* KT2440	MC	Ferulic acid, p-CA	Δ*pcaHG*, Δ*catA*, Δ*catA2*; *aroY*, *vanAB*, *pobA*, *kpdB*	Catechol	1.55 g/L.	98.5 mol%	NR	Flask	Yield reported, titer unavailable	[15]
*E. coli*	MC	Catechol	Δ*iscR*; *catA*, *MAR*	Adipic acid	<1 g/L	18 mol%	NR	Flask	Low titer and yield	[151]
*E. coli*	MC	Guaiacol	*catA*, *bcER*, *gcoAB*	Adipic acid	~0.6 g/L	98% conversion	NR	Flask	Low product concentration	[146]
*P. putida* KT2440	MC	PCA, Catechol	Δ*pcaF*, Δ*paaJ*; *PaaH*, *PaaF*, *Ter*	Adipic acid	2.5 g/L	17.4–18.4 mol%	NR	Fermenter	Low carbon yield	[51]
*P. putida* KT2440	MC	Catechol	Δ*catB*, Δ*catC*; *catA*	c,c-MA	39.9 g/L	95 mol%	NR	42.5-L fermenter	Benzoate/catechol-derived process	[152]
*P. putida* KT2440	MC	Ferulic acid, p-CA, Guaiacol	Δ*pcaHG*, Δ*catB*, Δ*catC*; *aroY*, EcdB, EcdD	c,c-MA	NR	~50 mol%	NR	Flask	Moderate yield	[38]
*P. putida* KT2440	Mixed (MC + AL)	Vanillate/PHB/lignin hydrolysate	Δ*pcaHG*, Δ*catB*; *pdc*, *kpdB*, *catA*	c,c-MA	NR	1–12% conversion	NR	Flask	Poor conversion from lignin hydrolysate	[143]
*P. putida* KT2440	Mixed (MC + AL)	Ferulic acid, p-CA, lignin	Δ*crc*; *aroY*, EcdB, EcdD	c,c-MA	50 g/L (MC); 4 g/L (AL)	100% conversion	>0.5 g/L/h	Fed-batch	Large drop with real lignin	[48]
*Sphingobium sp.* SYK-6	MC	Acetovanillone	Δ*pcaHG*, Δ*catB*; *acvABCDEF*, *vceAB*, *aroY*	c,c-MA	NR	~96% conversion	NR	Flask	Model substrate only	[144]
*N. aromaticivorans* DSM12444	AL	Alkaline pretreatment liquor	Δ*ligAB1*, Δ*ligAB2*, Δ*ligC*; *aroY*, *catA*	c,c-MA	NR	~100% from PCA equivalents	NR	Flask	Complex feedstock variability	[122]
*P. putida* KT2440	Mixed (MC + AL)	Guaiacol/kraft lignin fraction	Δ*catB*, Δ*catC*; *P450*, *ferredoxin*	c,c-MA	mM scale	~100% mol/mol	NR	Flask	Low titer	[50]
*C. glutamicum* MA-9	MC	Ferulic acid	Δ*fudC*, Δ*pcaHG*, Δ*catB*; *aroY*, EcdB, EcdD	c,c-MA	NR	100 mol%	NR	Flask	Titer not reported	[121]
*C. glutamicum*	Mixed (MC + AL)	Catechol/lignin hydrolysate	Δ*catB*; *catA*	c,c-MA	85 g/L (MC); 1.8 g/L (AL)	100 mol%	2.4 g/L/h	Fed-batch	Large performance gap between MC and AL	[153]
*P. putida* KT2440	Mixed (MC + AL)	Catechol/softwood lignin	Δ*catB*, Δ*catC*; *catA*, *catA2*	c,c-MA	64.2 g/L (MC); 13 g/L (AL)	100%	NR	Pilot scale	Lower titer from lignin	[123]
*Amycolatopsis sp* ATCC 39116	Mixed (MC + AL)	Guaiacol/pine lignin hydrolysate	Δ*catB1*, Δ*catB2*	c,c-MA	3.1 g/L	96 mol%	NR	Flask	Moderate titer	[154]
*P. putida* KT2440	MC	G-, H-, and S-aromatics	Δ*pcaHG*, Δ*catBC*; *aroY*, EcdB	c,c-MA	13.1 mM	99.5% yield	NR	Flask	Synthetic aromatic mixture, not lignin	[97]
*Sphingobium* sp. SYK-6	MC	Ferulic acid, p-CA	Δ*catA/A2*, Δ*pcaHG*; *vanAB*, *aroY*	Catechol	NR	36.3 mol%	NR	Flask	Model compounds only	[155]

* Feedstock Type: MC—lignin-derived model compound; AL—authentic lignin stream (lignin hydrolysate, black liquor, alkaline pretreatment liquor, kraft lignin fraction, etc.); NR: Not Reported.

## 10. Crossing the Cellular Barrier: Transport Engineering for Aromatic Uptake and Retention

**Figure 7:**
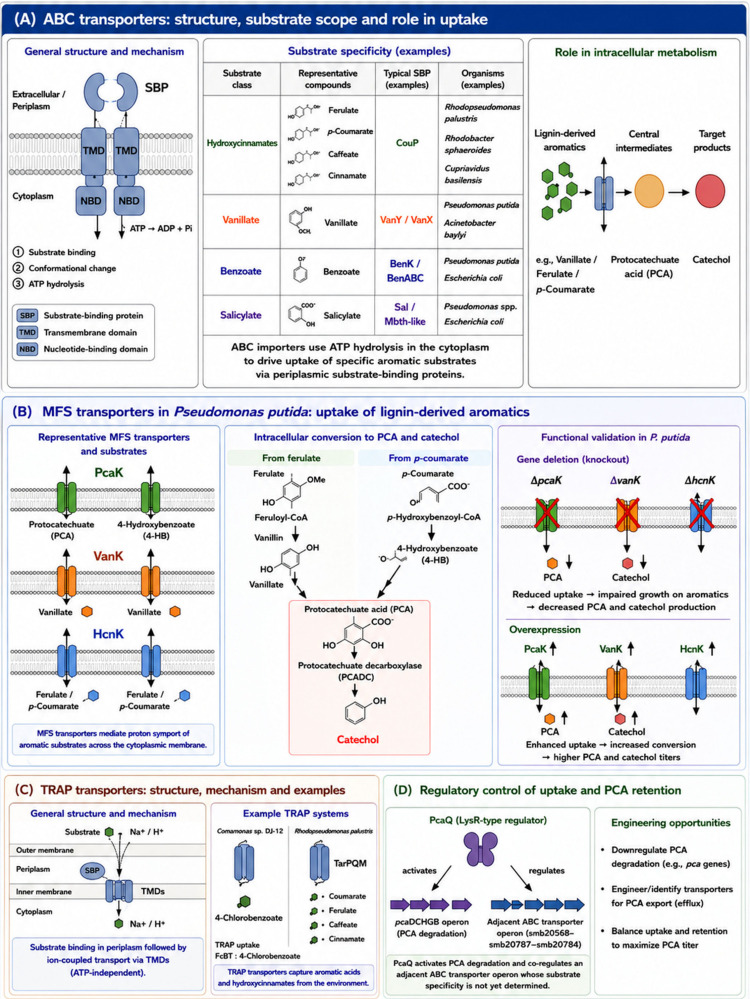
Transport engineering strategies for enhancing microbial uptake and conversion of lignin-derived aromatics to protocatechuic acid (PCA) and catechol. (**A**) ATP-binding cassette (ABC) transporters mediate ATP-dependent uptake of aromatic compounds through substrate-binding proteins (SBPs), with representative substrate specificities and their role in supplying intracellular metabolic pathways. (**B**) Major facilitator superfamily (MFS) transporters involved in lignin-derived aromatic uptake in *Pseudomonas putida*, illustrating representative transporters (PcaK, VanK, and HcnK), intracellular conversion of ferulate and *p*-coumarate to PCA and catechol, and the effects of transporter deletion or overexpression on aromatic assimilation and product formation. (**C**) Tripartite ATP-independent periplasmic (TRAP) transporters, showing their ion gradient-driven transport mechanism, representative systems, and uptake of hydroxycinnamate substrates. (**D**) Regulatory role of the LysR-type regulator PcaQ in controlling the *pcaDCHGB* operon and an adjacent ABC transporter gene cluster, together with engineering strategies to balance aromatic uptake, PCA retention, and intracellular conversion. Adopted from a previous study [65].

Following depolymerization, it is necessary to effectively transport lignin-derived aromatics across cell membranes for subsequent intracellular metabolism. The process by which microorganisms uptake lignin-derived aromatics has been increasingly revealed. Various aromatic molecules, including ferulic acid and pCA, were employed as substrates to generate PCA, catechol, and other chemicals. Engineering the transporter proteins is essential to increase aromatic substrate uptake and create the most efficient bacterial cell factories for PCA and catechol production. The transporters responsible for the uptake of aromatic compounds are primarily classified into three categories based on their transport mechanisms: ATP-binding cassette (ABC) transporters, major facilitator superfamily (MFS) transporters, and tripartite ATP-independent periplasmic (TRAP) transporters (Figure 7) [156].

ABC transporters are the most common and well-explored system for the uptake of different aromatic compounds’ energy from ATP hydrolysis to transport aromatics. It consists of transmembrane domains on the cytoplasmic side of the bacterial membrane, conserved nucleotide-binding domains, and an active substrate-binding region (SBR). The ABC transporters mediate the uptake of diverse aromatic compounds through substrate-specific binding proteins. CouP-type substrate-binding proteins from *Rhodopseudomonas palustris* have been experimentally shown to bind lignin-derived hydroxycinnamates, including coumarate, ferulate, caffeate, and cinnamate, whereas transport of other aromatic compounds depends on the specific ABC transporter involved [133,134]. CouP is a substrate-binding ABC transporter protein involved in the uptake of lignin-derived vanillin to produce catechol through PCA in *E. coli*. Overexpression of this protein significantly increased the catechol yield [120]. In *Rhodopseudomonas palustris*, this protein has been characterized for its binding affinity with ferulic acid. This high-affinity binding protein maximizes ferulic acid uptake [134].

Overexpression of this enzyme in the host cell could facilitate increased uptake of the lignin-derived aromatic for the efficient production of PCA and catechol. Identifying the specific transporters for PCA and catechol is important to avoid further degradation, while bacterial cell factories are engineered to produce these chemicals. In a previous study [128], a LysR protein named PcaQ was identified to bind to a specific DNA sequence upstream of the *pcaDCHGB* operon in *Sinorhizobium meliloti*. This operon encodes enzymes for PCA degradation. Interestingly, PcaQ also regulates another gene cluster (smb20568-smb20787-smb20786-smb20785-smb20784) in *S. meliloti*. This cluster is predicted to encode an ABC transporter system, a protein complex not previously known to transport PCA [157]. Suppressing these transporter proteins is important to stop the uptake of the PCA.

The MFS transporters constitute a substantial collection of secondary active transporters. Several MFS transporters have been studied, and their functions have been described. Examples include *PcaK*, a high-affinity transporter for protocatechuate (PCA) and 4-hydroxybenzoate in *P. putida*, 3-(3-hydroxyphenyl)propionate transporter MhpT from *E. coli* K-12, and gallic acid/PCA transporter GalT from *P. putida* [132,156]. The *P. putida* model strain possesses a transporter system that enables the uptake of various aromatics from lignin. The MFS transporters PcaK, VanK, and HcnK in *P. putida* are associated with the uptake of protocatechuate/4-hydroxybenzoate, vanillate, and ferulic acid/*p*-coumarate, respectively. Recent proteomic analyses of *P. putida* KT2440 demonstrated that aromatic substrate utilization is accompanied by substantial upregulation of transport proteins, including *HcnK*, *VanK*, and *PcaK*. The strong induction of these transporters during growth on lignin-derived phenolics suggests that membrane transport is a critical determinant of aromatic assimilation efficiency and represents an attractive target for transporter engineering to enhance lignin valorization [116]. Deleting these genes led to a significant decrease in the growth rate when PCA, vanillin, and ferulic acid/p-CA were the only carbon sources [125]. The *PcaK, Vank*, and *HcnK* proteins are crucial for the absorption of vanillin and ferulic acid/p-CA to generate PCA and catechol. The overexpression of *PcaK* in *P. putida* KT2440 resulted in an enhanced consumption of p-CA, leading to the increased synthesis of 2-pyrone-4,6-dicarboxylic acid [139].

TRAP transporters are an extensively researched group of secondary prokaryote transporters that rely on substrate-binding proteins. These are unidirectional secondary transporters that depend on Na+ or H+ gradients. TRAP transporters are composed of a substrate-binding protein and two transmembrane domains. TRAP transporters play a role in the absorption of 4-chlorobenzoate in *Comamonas* sp. Because 4-chlorobenzoate is not a lignin-derived aromatic, this transporter serves as a representative aromatic-acid TRAP transporter rather than a lignin-specific uptake system [130]. The TarPQM system in *R. palustris* plays a crucial role in absorbing and transforming aromatic compounds produced from lignin, such as coumarate, ferulic acid, caffeate, and cinnamate [134]. Various transporter proteins involved in the uptake of aromatic compounds in bacteria have been examined. Increasing the expression of the transporters for ferulic acid, pCA, vanillin, and phenols can enhance the synthesis of PCA and catechol. Furthermore, comprehending the arrangement and operation will aid in clarifying the absorption processes of aromatic compounds, which will direct the development of these carriers to enhance the efficiency of bacterial absorption of lignin-derived aromatic compounds.

While the uptake transporters discussed above govern the supply side of lignin aromatic metabolism, efflux pumps are equally important for the product side—particularly when the target compound is cytotoxic at production-relevant concentrations. Both PCA and catechol inhibit bacterial growth at millimolar levels [129]; catechol, in particular, intercalates into lipid membranes and disrupts membrane integrity. Resistance-nodulation-division (RND) family efflux systems are the best-characterised exporters of aromatic solutes in Gram-negative hosts. In *P. putida* DOT-T1E, three RND pumps—TtgABC, TtgDEF, and TtgGHI—act synergistically to confer tolerance to aromatic solvents including toluene, phenol, and catechol; TtgGHI alone is inducible by more than 30 aromatic compounds, including catechol and naphthalene derivatives [131]. Inactivation of TtgABC in *P. putida* has been shown to alter phenol tolerance, illustrating how pump expression levels can be tuned to balance product export against cellular fitness [158]. In *E. coli*, the AcrAB-TolC system is a broad-spectrum RND pump that exports phenolic and aromatic acid substrates; its overexpression has been used to improve tolerance to toxic bioproduction intermediates [159]. Similarly, the MexAB-OprM system of *Pseudomonas aeruginosa* accommodates a wide range of aromatic substrates, and its structural homology to the Ttg pumps suggests it as a candidate for heterologous expression in production strains [126]. Beyond RND pumps, ATP-binding cassette (ABC) and major facilitator superfamily (MFS) exporters have been implicated in aromatic tolerance in various *Pseudomonas* and *Rhodococcus* strains [160].

From an engineering standpoint, three strategies are emerging for harnessing efflux to improve PCA and catechol production: (i) overexpression of native RND pumps (e.g., TtgGHI) to increase catechol export and relieve product inhibition; (ii) heterologous expression of broad-spectrum pumps (e.g., AcrAB-TolC) in *P. putida* KT2440, which natively lacks TtgDEF and TtgGHI; and (iii) coupling efflux pump expression to product-responsive biosensors to create dynamic export circuits that activate only when intracellular product concentrations become inhibitory. Such strategies complement the uptake-engineering approaches described above and are essential for achieving the high titers required for economically viable PCA and catechol bioprocesses.

## 11. Downstream Processing and Techno-Economic Feasibility

### 11.1. Product Recovery and Purification

**Figure 8:**
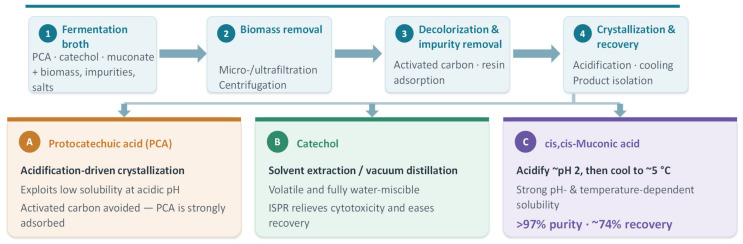
Representative downstream processing strategies for recovery of protocatechuic acid (PCA), catechol, and *cis,cis*-muconic acid. The workflow includes fermentation, biomass removal, impurity removal, and product recovery. (**A**) PCA is recovered by acidification-induced crystallization owing to its low solubility at acidic pH. (**B**) Catechol is typically recovered by solvent extraction and vacuum distillation, with in situ product removal (ISPR) helping to reduce product toxicity. (**C**) *cis,cis*-Muconic acid is recovered by acidification (pH ~2) and cooling-induced crystallization, achieving high purity and recovery.

Although most engineering effort has focused on improving titer, rate, and yield, the recovery of PCA and catechol from fermentation broth is itself a major determinant of process economics and is widely regarded as a bottleneck in lignin valorization. Aromatic products must be separated from residual biomass, unconverted aromatics, colored lignin-derived impurities, and the salts introduced during pH control. Typical recovery trains therefore combine biomass removal (micro-/ultrafiltration or centrifugation), decolorization and impurity removal by activated-carbon or resin adsorption, and final crystallization (Figure 8). For the closely related diacid *cis,cis*-muconic acid, acidification to ~pH 2 followed by cooling to ~5 °C exploits the strong pH- and temperature-dependent solubility of the molecule to crystallize the product at high purity (>97%) with ~74% recovery after activated-carbon polishing [48,49].

The same principles apply to PCA and catechol but with important caveats. PCA is itself strongly adsorbed by activated carbon—indeed, activated-carbon treatment is used to strip PCA from muconate streams—so PCA recovery relies primarily on acidification-driven crystallization, taking advantage of its low solubility at acidic pH. Catechol, being volatile and fully water-miscible, is more readily recovered by solvent extraction or vacuum distillation; moreover, because catechol is cytotoxic to the producing strain, in situ product removal (ISPR) by adsorption or extraction can simultaneously relieve product inhibition and simplify downstream recovery [161].

Catechol recovery imposes specific process constraints that differentiate it from PCA recovery and that have direct implications for downstream economics. Because catechol oxidises rapidly at elevated temperatures and in the presence of oxygen—forming the same *ortho*-benzoquinone responsible for its in vivo toxicity—distillation must be performed under reduced pressure (typically 10–50 mbar; boiling point ~80–110 °C under vacuum) and under nitrogen blanket to prevent quinone formation and product loss [161]. The recovery trains demonstrated for catechol therefore comprise four sequential operations: (i) cell separation by micro-/ultrafiltration or centrifugation; (ii) liquid–liquid extraction into a moderately polar organic solvent (methyl isobutyl ketone, ethyl acetate, or butyl acetate), achieving partition coefficients of 2–8 in favour of the organic phase; (iii) solvent recovery by vacuum distillation; and (iv) final purification by crystallisation from cold petroleum ether or by fractional vacuum distillation, yielding crystalline catechol at the ≥99% purity required for downstream polymerisation applications [161]. For downstream use as a *cis,cis*-muconate precursor, lower purity grades (90–95%) are tolerated, and integrated bioconversion—in which no catechol isolation step separates the catechol fermentation from the muconate-producing reactor—is increasingly favoured because it avoids the recovery train altogether and eliminates the oxidative-loss risk inherent in vacuum distillation [49,50,134]. The choice between high-purity isolation and integrated downstream coupling is one of the most consequential early process-design decisions for catechol biomanufacturing and should be driven by the target end-product portfolio rather than by analogy to the recovery trains of less reactive aromatics. Across all of these products, dilute titers and the salt burden generated by neutralization remain the principal cost drivers; integrated strategies such as high-pH fed-batch operation to reduce salt loading and ISPR to mitigate toxicity are therefore key levers for improving overall recovery economics [48].

The stringent recovery requirements for catechol arise directly from its intrinsic chemical reactivity, which also underlies its pronounced cytotoxicity during microbial production. Consequently, improving intracellular tolerance is as important as optimizing downstream processing for achieving economically viable catechol biomanufacturing. Catechol toxicity results from membrane disruption, ROS generation, quinone-mediated protein damage, and depletion of intracellular glutathione, with concentrations above 5–10 mM severely inhibiting bacterial growth [108,129,158,162]. To improve tolerance, engineered strains overexpress *gshA*/*gshB*, quinone reductases, molecular chaperones, and efflux pumps, while adaptive laboratory evolution (ALE) introduces beneficial mutations that further enhance resistance [55,159,160,162] At the bioprocess level, in situ product removal (ISPR) maintains extracellular catechol below toxic levels and, when combined with biosensor-driven dynamic control, offers the most promising strategy for achieving industrially relevant catechol titres (>30 g L^−1^) [29,30,31,32].

### 11.2. Techno-Economic and Life-Cycle Assessment

**Figure 9:**
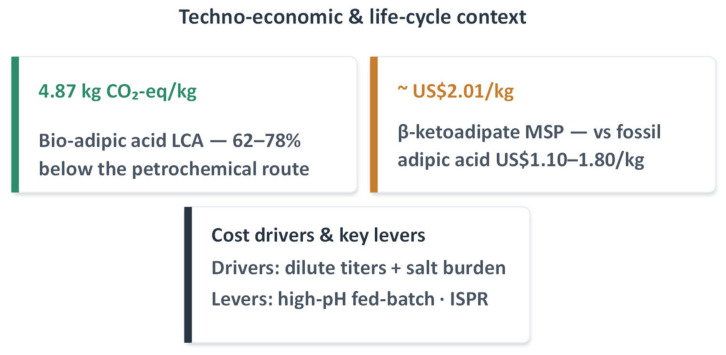
Techno-economic and life-cycle considerations for lignin-derived aromatic bioprocesses. Representative life-cycle assessment (LCA) and techno-economic analysis (TEA) metrics for bio-based adipic acid production are shown alongside the major cost drivers and engineering strategies influencing process economics, including titer improvement, salt reduction, high-pH fermentation, and in situ product removal (ISPR).

Dedicated techno-economic analyses (TEA) and life-cycle assessments (LCA) of microbial PCA or catechol production are still scarce; most quantitative assessments instead target the downstream diacids—muconic acid, adipic acid, and β-ketoadipate—for which PCA and catechol are the immediate biosynthetic precursors. These studies nonetheless bound the economic and environmental performance of the entire funneling route. An LCA of bio-based adipic acid produced from lignin estimated greenhouse-gas emissions of 4.87 kg CO_2_-eq per kg of adipic acid—a 62–78% reduction relative to the petrochemical route—driven largely by avoided nitrous-oxide emissions and valorization of a biorefinery side-stream rather than its combustion (Figure 9) [147]. A complementary limited LCA and cost assessment of the lignin-to-adipic-acid chain (via catechol and muconate) similarly projected substantial reductions in CO_2_-equivalent emissions and energy demand for the bio-based process, while identifying lignin depolymerization selectivity and biocatalyst toxicity as the critical levers for feasibility [152]. On the cost side, a TEA of β-ketoadipate produced directly from lignin-related aromatics in *P. putida* estimated a minimum selling price of ~US$2.01 per kg—competitive with fossil-derived adipic acid (~US$1.10–1.80 per kg)—provided that high titers, rates, and yields are maintained at scale [124].

Two themes emerge consistently from these analyses and are directly relevant to PCA/catechol biomanufacturing. First, process economics are dominated by the bioconversion metrics (titer, rate, and yield) together with feedstock cost and the selectivity of the upstream depolymerization, which governs both substrate availability and inhibitor load. Second, partial conversion of lignin to defined chemicals is environmentally preferable to its combustion for heat and power, reinforcing the rationale for the engineering strategies discussed in this review. The current absence of TEA/LCA studies focused specifically on PCA and catechol represents a clear research gap; closing it will be essential to translate the laboratory advances summarized here into economically and environmentally viable processes.

## 12. Conclusion and Future Research Challenges

In this review we have argued that the field of microbial lignin valorization to aromatic platform chemicals is best organised around a single conceptual unit—the *PCA*–catechol junction—and we have used the three principles of this framework (convergence, switch, divergence) as the design unit through which biofunneling pathways, PCADC engineering, transporter engineering on both sides of the membrane, and downstream processing have been examined. The framework reduces an apparently disparate engineering problem to a small number of moves at one decision point: block *pcaHG*/*ligAB* to accumulate PCA; introduce PCADC plus its accessory factors to bridge to catechol; block *catA*/*catA2* and *catBC* to accumulate catechol; or proceed to the target downstream product. Within this framework, three classes of open problem now define the research agenda, mapped to the three principles of the junction: (i) for convergence, systematic bioprospecting and adaptive laboratory evolution to expand the demethylase and ligninolytic enzyme repertoires available to engineering chassis; (ii) for the switch, rational engineering of PCADC activity and accessory-protein reconstitution to relieve the rate-limiting step at the centre of the framework; and (iii) for divergence, relief of carbon catabolite repression (e.g., Crc) and engineering of efflux pump–biosensor circuits to mitigate product toxicity. We submit that organising the field around the junction—rather than around individual end-products—is the conceptual shift required to translate the laboratory advances summarised here into industrially viable processes. Lignin valorization offers a compelling route to PCA and catechol, yet bacterial conversion remains challenging. This review surveys metabolic engineering strategies for producing PCA, catechol, and their derivatives via ring-cleavage pathways. Notably, most reported strains act on purified lignin-derived aromatics rather than lignin itself, underscoring the need for bacteria that combine lignin depolymerization with aromatic assimilation.

Systematic bioprospecting from environments with high natural ligninolytic pressure represents the most direct route to discovering such strains. Termite guts harbour specialised bacterial communities—dominated by Spirochaetes, Firmicutes, and Proteobacteria—that achieve lignocellulose hydrolysis efficiencies exceeding 90%, and shotgun metagenomic sequencing of these consortia has already yielded novel glycoside hydrolases and peroxidases with confirmed lignin-degrading activity. Compost microbiomes, particularly during thermophilic stages, harbour actinobacterial and *Bacillales* lineages with strong ligninolytic enzyme production; time-series metatranscriptomics has revealed that lignin deconstruction occurs sequentially and synergistically with hemicellulose degradation, and has enabled near-complete genome reconstruction of novel biodegrading species. Pulp-mill effluents and kraft black-liquor streams, chronically enriched in lignin-derived aromatics, are likewise productive sources: strains of *Pseudomonas*, *Rhodococcus*, and *Serratia* isolated from such sites routinely achieve 70–80% lignin removal. Integrating 16S rRNA amplicon profiling with shotgun metagenomics and functional screening (e.g., dye-decolorising peroxidase assays, milled-wood-lignin plate clearance) provides a robust pipeline for moving from environmental sample to candidate chassis in a targeted manner. These approaches complement the metabolic-engineering strategies discussed in this review by expanding the pool of naturally competent hosts that combine lignin depolymerization, aromatic funneling, and tolerance traits within a single genome.

Recent advances in transcription factor-based, whole-cell, and nucleic acid-based biosensors have enabled real-time monitoring of lignin-derived aromatic intermediates, high-throughput screening of engineered enzymes and strains, and dynamic regulation of metabolic pathways. In parallel, lignin-derived nanomaterials, including lignin nanoparticles, quantum dots, and nanozymes, are gaining attention as sustainable biosensing platforms. The combination of biosensor-guided pathway optimization, machine learning, and synthetic biology is expected to accelerate the development of next-generation microbial platforms for efficient production of PCA, catechol, and other value-added aromatic compounds from renewable lignin feedstocks.

Future research should focus on improving the catalytic activity, pathway flux, and cofactor regeneration of the enzymes that convert lignin-derived intermediates into PCA and catechol. Another main challenge in lignin valorization for PCA and catechol production is substrate toxicity to the host strain. It is well-reported that PCA and catechol have growth-inhibitory effects on different bacteria. Engineering bacterial strains to resist PCA and catechol toxicity is crucial for improving their production. Introducing genes for PCA and catechol efflux proteins into these hosts enables the continuous removal of these compounds from the cells. This reduces intracellular accumulation and mitigates the toxic effects of PCA and catechol on the bacteria. Although substantial advances have been made in engineering microbial pathways for PCA and catechol production, many reported studies rely on purified lignin-derived aromatic compounds rather than directly utilizing heterogeneous lignin streams. Bridging this gap remains a critical challenge for the field, requiring the integration of efficient lignin depolymerisation technologies with robust microbial conversion platforms that can tolerate complex lignin-derived feedstocks.

## Figures and Tables

**Table 1:** Comparison of microbial chassis used for PCA and catechol production from lignin-derived aromatics. Citations refer to the main reference list.

Host	Native Aromatic Catabolism	prFMN/PCADC Compatibility	Aromatic/Product Tolerance	Genetic Tractability	Industrial Verdict
*P. putida* KT2440	Native β-ketoadipate pathway; H/G aromatics fully covered [117,118]	Native UbiX-like activity; sufficient endogenous prFMN [15]	High; solvent-tolerant membrane (Ttg pumps) [133,134]	Mature CRISPR/genetic toolkit [55].	Present leader for native lignin streams [15,46]
*C. glutamicum*	Limited; requires extensive engineering [130]	Incomplete prFMN/PCADC system; reconstruction needed [130]	Highest of the four; robust industrial fermentation [130]	Established industrial toolkit	Most attractive future chassis if UbiX gap is closed [130,135]
*E. coli*	Absent; shikimate-derived PCA or full cassette import needed [136]	Native UbiX dedicated to ubiquinone; full KpdBCD/EcdBD required	Low; catechol bactericidal at low mM [116]	Unparalleled [136]	Proof-of-concept workhorse; restricted industrial applicability
*Sphingobium* sp. SYK-6	Specialized; unmatched S-lignin 4,5-cleavage [96,97]	Not fully characterized	Moderate [125]	Limited [125]	Indispensable for syringate; impractical for large scale

## Data Availability

No new data were created or analyzed in this study. Data sharing is not applicable to this article.

## Abbreviations

ABC	ATP-Binding Cassette
*acvABCDEF*	Acetovanillone Synthetase Gene Cluster
ATP	Adenosine Triphosphate
*catA*	Catechol 1,2-Dioxygenase
*catA*/*A2*	Catechol 1,2-Dioxygenase Isozymes
*catB*	Muconate Cycloisomerase
*catC*	Muconolactone Isomerase
cc-MA/c,c-MA	*cis,cis*-Muconic Acid
CoA	Coenzyme A
CRISPR	Clustered Regularly Interspaced Short Palindromic Repeats
DyP	Dye-Decolorizing Peroxidase
ECH	Enoyl-CoA Hydratase/Lyase
FCS	feruloyl-CoA synthetase
VDH	vanillin dehydrogenase
FDC	ferulic acid decarboxylase
VGDH	4-vinylguaiacol dehydrogenase
DCL	decarboxylase
V-O-DML	vanillate O-demethylase
pHCS	p-hydroxycinnamoyl-CoA synthetase
pHCHL	p-hydroxycinnamoyl-CoA hydratase/lyase
BADH	benzaldehyde dehydrogenase
pHBDC	p-hydroxycinnamic acid decarboxylase
pHB3H	p-hydroxybenzoate 3-hydroxylase
DesA	syringate O-demethylase
DesV	syringate decarboxylase
DesZ	3-O-methylgallate 3,4-dioxygenase
LigM	vanillate/3-O-methylgallate O-demethylase
LigAB	protocatechuate 4,5-dioxygenase
LigC	4-carboxy-2-hydroxymuconate-6-semialdehyde dehydrogenase
LigI	2-pyrone-4,6-dicarboxylate hydrolase
LigJ	2-keto-4-carboxy-3-hexenedioate hydratase
BenAB	benzoate 1,2-dioxygenase
BenD	dihydrodiol dehydrogenase
SH	salicylate 1-hydroxylase
VDC	vanillate decarboxylase
P450-O-DML	cytochrome P450 monooxygenase (O-demethylation)
PCADC	protocatechuate decarboxylase
C23O (C-2,3D)	catechol 2,3-dioxygenase
HMSD	2-hydroxymuconic semialdehyde dehydrogenase
HMD	2-hydroxymuconic acid decarboxylase
OCT	4-oxalocrotonate tautomerase
OPH	2-oxopent-4-enoate hydratase
HMSH	2-hydroxymuconic semialdehyde hydrolase
HAO	4-hydroxy-2-oxovalerate aldolase
FA	Ferulic Acid
G-ligni	Guaiacyl Lignin
HcnK	Hydroxycinnamate Transporter
H-lignin	p-Hydroxyphenyl Lignin
LiP	Lignin Peroxidase
LRC	Lignin-Related Compounds
MAR	Medium-Chain Alcohol Dehydrogenase/Reductase
MFS	Major Facilitator Superfamily
MnP	Manganese Peroxidase
NAD(P)H	Reduced Nicotinamide Adenine Dinucleotide (Phosphate)
NADH	Nicotinamide Adenine Dinucleotide (Reduced Form)
NF-κB	Nuclear Factor Kappa B
P450	Cytochrome P450 Monooxygenase
pCA	p-Coumaric Acid
PCA	Protocatechuic Acid
PDC	2-Pyrone-4,6-Dicarboxylic Acid
pHBA	p-Hydroxybenzoic Acid
PI3K/Akt	Phosphoinositide 3-Kinase/Protein Kinase B
ROS	Reactive Oxygen Species
SBR	Substrate-Binding Region
S-lignin	Syringyl Lignin
TCA Cycle	Tricarboxylic Acid Cycle
TRAP	Tripartite ATP-Independent Periplasmic Transporter
*vanAB*	Vanillate O-Demethylase
VanS	Vanillin Synthase
β-KA/β-KAP	β-Ketoadipate
